# Estimating the Diets of Animals Using Stable Isotopes and a Comprehensive Bayesian Mixing Model

**DOI:** 10.1371/journal.pone.0028478

**Published:** 2012-01-03

**Authors:** John B. Hopkins, Jake M. Ferguson

**Affiliations:** 1 Department of Ecology, Montana State University, Bozeman, Montana, United States of America; 2 Department of Biology, University of Florida, Gainesville, Florida, United States of America; University of Illinois at Champaign-Urbana, United States of America

## Abstract

Using stable isotope mixing models (SIMMs) as a tool to investigate the foraging ecology of animals is gaining popularity among researchers. As a result, statistical methods are rapidly evolving and numerous models have been produced to estimate the diets of animals—each with their benefits and their limitations. Deciding which SIMM to use is contingent on factors such as the consumer of interest, its food sources, sample size, the familiarity a user has with a particular framework for statistical analysis, or the level of inference the researcher desires to make (e.g., population- or individual-level). In this paper, we provide a review of commonly used SIMM models and describe a comprehensive SIMM that includes all features commonly used in SIMM analysis and two new features. We used data collected in Yosemite National Park to demonstrate IsotopeR's ability to estimate dietary parameters. We then examined the importance of each feature in the model and compared our results to inferences from commonly used SIMMs. IsotopeR's user interface (in R) will provide researchers a user-friendly tool for SIMM analysis. The model is also applicable for use in paleontology, archaeology, and forensic studies as well as estimating pollution inputs.

## Introduction

Stable isotopes were first used to investigate the foraging ecology of animals in the 1970s [1]–[5]. Early studies used stable isotope analysis (SIA) to determine the relative importance of food sources to animals by comparing distributions of isotope ratios (expressed as isotope values; derived below) for animal tissues to the foods they consume after corrected for fractionation (the sorting of isotopes during natural biochemical processes)—a technique primarily used when food sources had distinctly different isotope values (e.g., C_3_ and C_4_ plants, or prey that differ in trophic level) [2], [6]. Isotope values (e.g., ™X, ™Y) are expressed in delta (™) notation as per mil (‰) units (or parts per thousand):
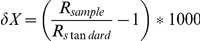
where R is the ratio of heavy to light isotopes (e.g., ^13^C/^12^C or ^15^N/^14^N) in the sample and the standard [7]. Samples with a lower ratio of heavy isotopes relative to the standard will yield a negative value and samples with higher ratios will have a positive value.

For the past few decades, SIA has gained popularity among ecologists (e.g., [8]–[13]). In particular, stable isotope mixture models (often called mixing models; hereinafter SIMMs) are commonly used to estimate the relative contribution of assimilated dietary sources to the tissues of animals (i.e., the conversion of food nutrients into tissues by the processes of digestion and absorption), and if certain assumptions are met (Table 1), the diets of animals. Euclidian distance formulas were used in some early studies (e.g., [14]–[18]); however, these methods did not provide correct solutions for observed and simulated data [19]. Specifically, these Euclidean distance models failed to preserve mass balance, an application of the law of conservation of mass which states that the proportional assimilated dietary contributions (mass) flowing into an organism or population are constrained to sum to one. Recently, variants of mass-balance models have developed rapidly [19], [20]. Although the profusion of SIMMs (many of which are discussed in this paper) indicates the importance of this field to ecologists, current models require researchers to make tradeoffs (Table 1) when choosing one model over another.

**Table 1:** A comparison of SIMM assumptions and features among commonly used SIMMs.

Models	IsotopeR	SIAR	Semmens et al. 2009	MixSIR	IsoConc	IsoError	IsoSource
**SIMM assumptions:**							
*Elemental concentration (e.g., [C] and [N]) of all dietary items are equal*							
• Different source concentrations for dietary sources	X	X			X		
*Elements are assimilated with the same efficiency*							
• Different assimilation efficiencies for dietary sources	X	Y			Y		
*No tissue-diet discrimination*							
• Variation associated with predicted discrimination factors	X	X	X	X			
• Includes a fixed “discrimination error” term (calculated *a priori*): error associated with the regression model used to predict discrimination factors	X						
*No isotopic routing*							
• Differential allocation of isotopically distinct dietary sources to different tissues							
**Other SIMM features:**							
Uses a Bayesian analytical framework	X	X	X	X			
Uses a fully Bayesian approach	bX						
Sampling procedure used to estimate parameters	MCMC	MCMC	MCMC	SIR	ML	ML	ML
Uses raw data (not parameter estimates of raw data) to simultaneously estimate parameters (random variables): dietary sources (including isotopic correlation, variation), measurement error, proportional source contributions at the population- and individual-level	bX						
Measurement error: variation associated with SIA: sample preparation error and error during mass spectrometry; applied to each observation in the study	X						Y
Source process error: inherent isotopic variation of the sampled source (i.e., within and between individual plants and animals of the same species or taxa)	X	X	X	X		X	
Mixture process error: inherent isotopic variation in a sub-sampled tissue (e.g., non-homogenized hairs, feathers, claws from the same individual) and/or sample of mixtures (e.g., population)	X	X	X	X		X	X
Correlation of isotope values in sources: accounts for the linear relationship among isotope values for different elements	X					X	
A residual error term	X	X		X			
Individual-level source estimation using hierarchical design	X		X				
Prior information associated with sources (e.g., source proportions, distribution of isotope values, elemental concentrations) and mixtures (e.g., measurement error)	X	X	X	X			
Calculates proportional dietary source estimates when >n+1 sources	X	X	X	X			aX

Four mixing model assumptions (italics) commonly violated when estimating the proportional dietary contribution of sources to the diets of animals, and the model feature that addresses each violated assumption. A list of other features included in SIMMs and their definitions. X denotes the model addresses the assumption or includes the feature and Y indicates the feature is not explicitly included (e.g., model may account for error using an arbitrary tolerance measure). MCMC (Markov chain Monte Carlo), SIR (sequential importance resampling), and ML (maximum likelihood) denotes sampling method used when estimating parameters.

a X denotes that the model provides solutions when sources exceed *n*+1, but solutions are not comparable to other models (i.e., output lists ranges of potential solutions, not parameter estimates).

b X indicates Ward et al. (35) was the first study to use this approach. However, this model (35) has recently been introduced; therefore, it has not been commonly used.

All models discussed in this paper use the same basic methodology for estimating proportional source contributions to the diets of animals. For example, a duel element (X, Y), three-source, mass-balance, linear mixing model is described by the following equations [20]:
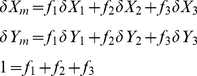
(1)This system of three equations yields three unknown proportional source contributions (ƒ_1_, ƒ_2_, ƒ_3_) for a mixture (m) when ™X and ™Y values are known for mixtures and sources (the latter adjusted to account for isotopic discrimination; described below).

In the following sections, we discuss the SIMMs commonly used to estimate dietary parameters and follow this review with details about our comprehensive SIMM, IsotopeR.

### Frequentist SIMMs

#### *IsoError*

Phillips and Gregg [21] refined the application of linear mass-balance procedures (equation set 1) with IsoError. This SIMM can be applied to systems where the number of sources do not exceed n+1 (n = number of isotope systems); however, when sources do exceed n+1, the system of equations is underdetermined and the model cannot be used. IsoError calculates deterministic solutions and allows a user the ability to incorporate the process error and the isotopic correlation in sources and mixtures (Table 1).

Isoerror does not address many of the assumptions (Table 1) that may be violated when estimating diets using SIMMs [19], [22], [23]. In addition, neither IsoError nor the mass balance equations (equation set 1) are constrained to yield proportional source contributions (*f* variables in equation set 1) in the interval (0,1). Therefore, when data fall outside the isotopic mixing space (the area or volume contained in the space formed by lines connecting the sources in multivariate isotope space) because an important food source was overlooked, the wrong discrimination factor was applied to a source, or a mixing model assumption was violated [24], nonsensical negative proportions are calculated for dietary contributions.

#### *IsoConc*

Most stable isotope mixing models assume that the elemental concentrations of dietary items are equal. Although this assumption is valid for many carnivore and herbivores, it is often violated for omnivores who feed on a variety of dietary sources at different trophic levels [24]. IsoConc was developed to estimate the contribution of each source to the diets of animals by assuming a source contribution is proportional to the assimilated biomass of the source multiplied by the elemental concentration (e.g., %C, %N) of the source [24]. This model was the first to transform a polygonal mixing space to a curved mixing space [24].

Standard linear mixing models and the examples presented in Phillips and Koch [24] assumed that all sources are equally digestible. In response, Robbins et al. [25] pointed out the need to consider digestibility when determining the elemental concentrations of sources. In reply, Koch and Phillips [26] calculated the digestibility of macronutrients in food sources and included the corrected elemental contributions of these sources in their diet estimation. By incorporating “concentration dependence” and explicitly including the digestibility of sources in their calculation, this SIMM made a significant stride towards estimating more accurate dietary parameters [26]. However, unlike IsoError, IsoConc does not allow the user to incorporate various sources of error inherent to SIMM analysis.

#### *IsoSource*

IsoSource was developed to calculate the frequency and range of potential source contributions in situations where the number of sources exceeds n+1 [27]. Using the standard linear mixing model, IsoSource systematically creates each combination of possible source contributions (that sum to 1.0) by a certain increment (e.g., 0.01). Next, the model predicts mixture isotope values for each combination using source isotope values (means). If these predicted values fall within a certain designated mass balance tolerance (e.g., ±0.1‰; which accounts for the error associated with measurement and source variability) then the combination is considered a feasible solution; Phillips and Gregg [27] suggested reporting the distribution of feasible solutions.

This model can be helpful at inferring possible diet compositions when a unique solution cannot be calculated, but it has limits when investigating many ecological questions [28]. In particular, each feasible solution is no more probable than another; therefore, the results are difficult to interpret—especially when the range of certain source proportions (minimum and maximum values selected from the solution set for a particular source) is wide (e.g., 0.1–0.9).

### Bayesian SIMMs

Bayesian SIMMs allow ecologists to fit probability models to isotopic data. These models can include various sources of uncertainty, greater than n+1 sources, prior information, and a hierarchical structure in a flexible and intuitive estimation framework. Specifically, these Bayesian models allow users to efficiently estimate numerous parameters while avoiding calculation of multidimensional derivatives, as in likelihood methods.

Several Bayesian SIMMs have been used to estimate proportional dietary contributions at the population- [29], [30] and individual-level [31]. The earliest model, MixSIR (v.1.0.4) [29], estimates the joint posterior probability of sources used by animals (reported as marginal distributions for each dietary source contribution) by importance sampling (less efficient sampling method than Markov chain Monte Carlo sampling) and incorporates the following isotopic information in the model: (1) source mean and standard deviation, (2) tissue-diet discrimination factor mean and standard deviation, (3) mixture data (single consumer or sampled population), and (4) a Dirichlet prior on the proportional estimators (recommended by Jackson et al. [32] and incorporated in Semmens et al. [31]). Although MixSIR may calculate reasonable dietary estimates in some cases, its credible intervals may be too narrow because the model does not account for variation among individuals and other sources of error (Table 1).

Currently, two other Bayesian SIMMs are commonly used [30], [31]. Semmens et al. [31] built the first hierarchical Bayesian model to account for intra-population variability in resource use when estimating the diet of a population (hereinafter Semmens et al. model). This model is very useful because it allows researchers to estimate diets at both the population- and individual-level. In general, hierarchical models are used to make such individual-level inference possible; however, difficulties may persist when estimating individual diets. Specifically, these hierarchical models use information from the population-level to estimate individual diets; therefore, when the population sample size is large, individual estimates will be pulled to the population mean [33]. Currently, it is unknown what the ideal sample size is for individuals when making individual-level inference. However, it is certain that the population has a major influence on individual diet estimates and repeated measures for individuals will improve inference [33].

Another model, the SIAR model [30]—originally developed as an R package [34] and first described by Jackson et al. [32]—allows a user to incorporate unequal elemental concentrations in sources when estimating the diets of animals at the population-level. Although these new Bayesian models provide reasonable estimates for proportional dietary contributions, they lack the ability perform an analysis that incorporates both concentration dependence and individual-level estimation simultaneously.

Here, we explore the assumptions associated with SIMM analysis, combine SIMM features (i.e., components of the model expressed in mathematical terms), and develop two new features for our comprehensive SIMM model called IsotopeR. We use the hierarchical model structure of Semmens et al. [31] and the concentration dependence formulation originally developed by Phillips & Koch [24] as the foundation for our model, while incorporating all other SIMM features to more accurately infer proportional diet compositions (Table 1). In addition, we use a fully Bayesian approach similar to Ward et al. (35) to jointly estimate parameters. Joint estimation is useful when estimating multiple dependent quantities because it accounts for the inherent uncertainty associated with the joint estimation process. Not accounting for this uncertainty can lead to overly precise credible intervals.

We validated IsotopeR by estimating the relative contribution of sources to the diets of male food-conditioned (FC; [36]) black bears (*Ursus americanus*) sampled in Yosemite National Park (YNP). Our purpose was to use real data to estimate dietary parameters using IsotopeR, not to accurately estimate the real diets of YNP black bears. We also examined the effect of each feature on inference by systematically removing them from the model independently. Lastly, we compared IsotopeR estimates to those from other frequently used models.

## Methods

### Sampling

#### Mixtures

Yosemite National Park Wildlife Management staff live-captured FC black bears primarily in Yosemite Valley for management purposes from August 2005 through September 2007 (Table S1). They captured and immobilized bears in culvert traps according to Park Service protocol. They collected bear tissues in accordance to Wildlife Management protocol. For hair, they collected ten or more full-length guard hairs from along the spines or upper limbs of bears during spring and early summer or from the lower limbs or flanks in late summer and fall. We assumed hairs collected during spring and early summer months were grown the previous year, whereas hairs collected in the fall were grown the current year [37].

#### *Sources*

We collected the following bear foods opportunistically in 2007 because they were identified by previous diet studies (i.e., fecal analysis) as being important natural food sources for bears throughout YNP [38], [39]: acorns (Quercus kelloggii, Quercus wislizenii), manzanita berries (Arctostaphylos spp.), grass (Agrostis spp.), forbs (Trifolium spp., Lupinus spp., Montia spp.), and animals [ants (Formicida), wasps (Vespidae), bees (Apidae), termites (Isoptera), and mule deer (Odocoileus hemionus)](Table S2). We used the isotope values for these foods to estimate the isotopic signature of natural sources (100% plant diet, 100% animal diet).

**Figure 1:**
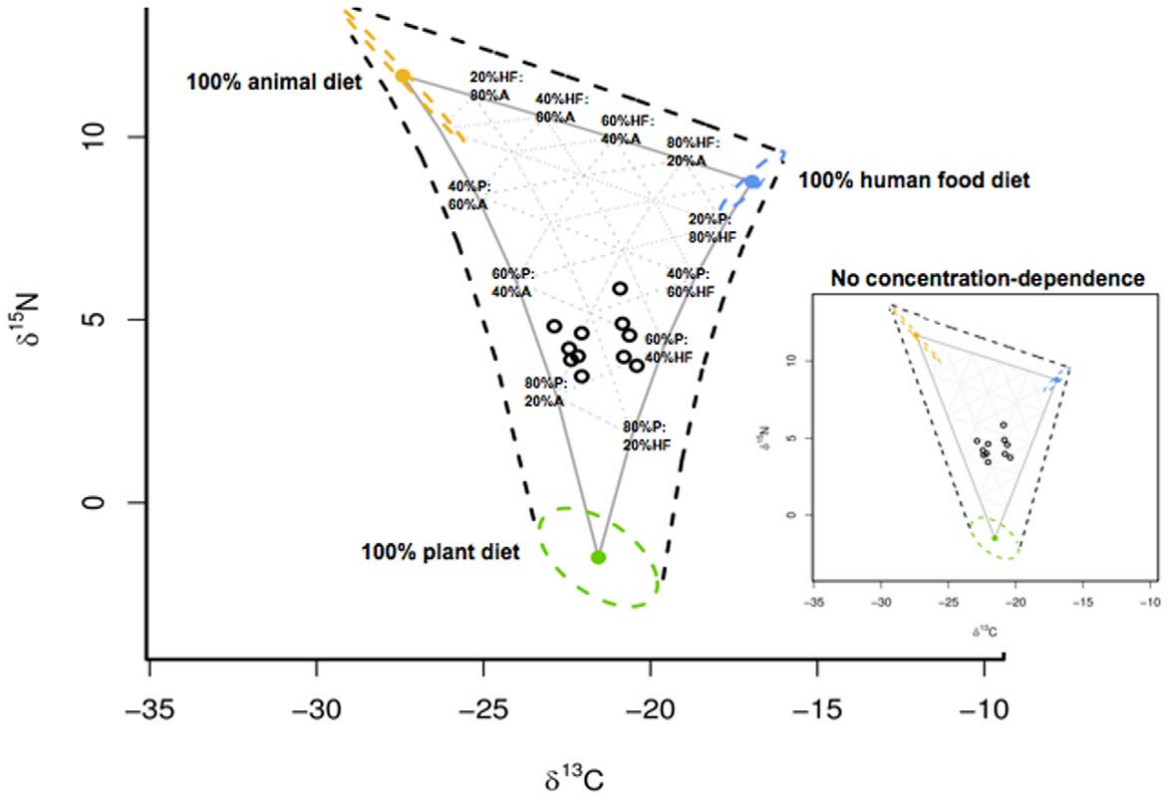
Isotopic mixing space for FC black bears sampled in Yosemite National Park. Isotope values (δ^13^C and δ^15^N) for male bears (open circles) captured in YNP and their estimated food sources. Estimated means for source aggregates (100% plant diet [green circle], 100% animal diet [orange circle], 100% human food diet [blue circle]) and process error (1 SD; dashed ovals) were estimated by IsotopeR and defined the vertices of the dietary mixing triangle; the shape of each source aggregate illustrates the degree of estimated isotopic correlation of observations used to define each source (see Fig. 4). Variations in dietary contributions (%) of plants (P), animals (A), and human food (HF) are shown along the edge of the mixing triangle (solid gray line) that connects estimated source means; labels denote the contribution of diet when consumers lie at the intersection of the mixing triangle edge and gray dashed iso-diet lines (within the triangle). The black dashed triangle illustrates the approximate total mixing space at 1 SD. Measurement error (not shown) was also estimated by IsotopeR and applied to each source observation when estimating source aggregates and to each bear in the mixing space. The inset illustrates the isotopic mixing space if concentration dependence was not included in the analysis.

We collected human hair samples in 2009 from floor clippings at two salons and one barbershop in St. Louis, MO (n = 20; Table S3); collecting these samples from the garbage did not require an ethics permit. We compared isotopic results from 2009 to results from a 2004 nation-wide survey of human hair (n = 52) [40]. We found that the two samples were isotopically indistinguishable (2004: δ^13^C (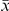) = −16.9±0.8, δ^15^N (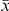) = 8.8±0.5; 2009: Table S3; t = −0.79, df = 71.62, P = 0.43); therefore, we pooled samples to form the human food aggregate (i.e., 100% human food diet; Fig. 1, Tables 2A & C). We assumed that bears on 100% human food diet would be isotopically similar to humans because both humans and bears are monogastric omnivores; thus, it is likely that they discriminate against ^14^N and ^12^C by a similar magnitude.

**Table 2:** Bear food sources.

Aggregate	δ^13^C (‰)	δ^15^N (‰)	*r*	Δ^13^C (‰)	Δ^15^N (‰)	%C	%N	Digest [C]	Digest [N]
**A. Frequentist models**	**Discrimination included:**								
Plants	−21.47 (2.83)	−1.48 (1.61)	−0.29			45.41 (3.92)	1.57 (1.03)	47.29 (3.43)	3.51 (3.09)
Animal	−27.44 (1.82)	11.71 (1.74)	−0.83			48.26 (3.81)	12.17 (1.69)	51.50 (0)	12.17 (1.69)
Human	−16.94 (0.79)	8.78 (0.47)	0.58					52.83 (2.54)	6.88 (1.10)
Bear	−21.60 (0.88)	4.37 (0.68)	0.17						
**B. IsotopeR estimates**									
Plants	−21.72 (2.66)	−1.42 (1.61)	−0.28			45.45 (3.94)	1.57 (1.03)	47.28 (3.91)	3.42 (2.28)
Animal	−27.43 (1.61)	11.69 (0.29)	−0.91			48.28 (3.86)	12.14 (1.70)	51.50 (0.06)	12.18 (1.63)
Human	−16.95 (0.29)	8.78 (0.27)	0.69			Fixed estimates (same as A)			
**C. Other Bayesian models**	**Discrimination separate:**								
Plants	−27.53 (2.25)	−0.75 (1.19)		6.06 (0.90)	−0.73 (0.90)	45.41 (3.92)	1.57 (1.03)	47.29 (3.43)	3.51 (3.09)
Animal	−24.23 (0.71)	3.16 (1.00)		−3.22 (1.48)	8.55 (1.48)	48.26 (3.81)	12.17 (1.69)	51.50 (0)	12.17 (1.69)
Human	−16.94 (0.79)	8.78 (0.47)		Discrimination included		Fixed estimates (same as A)			

A) Discrimination-corrected plant (n = 48), animal (n = 29), and human food (n = 72) sources (aggregates) calculated from the sample data. (B) Plant and animal sources estimated by IsotopeR. Human food concentrations are fixed as in A and C (see Table S4). (C) Raw isotope values and discrimination factors used in IsoSource and other Bayesian models. Mean and (1 SD) reported.

We estimated the elemental concentration ([C] and [N]) of the average human diet in the United States by analyzing nutrient data from the USDA National Nutrient Database (NDB: http://www.nal.usda.gov/fnic/foodcomp/search/; Table S4). First, we determined amount of digestible C and N in samples from each food group (n≥3 food items). Then we weighed the food group based on the fractional contributions of these food groups to the diets of humans [41]. Lastly, we used the weighted values to estimate the average digestible [C] and [N] for human foods (Table S4). We used these estimates to construct the isotopic mixing space used in our example diet analysis, and unlike the plant and animal aggregate, this aggregate was not estimated using Bayesian methods.

### Sample preparation, analysis, and Suess effect correction

We rinsed guard hairs with a 2∶1 chloroform-methanol solution to remove surface oils. We oven-dried plants and homogenized each sample. We then weighed all samples into tin cups (4×6 mm). The Stable Isotope Laboratory at University of California, Santa Cruz, CA analyzed samples for their carbon (δ^13^C) and nitrogen (δ^15^N) stable isotopic composition by continuous flow methods using a Carlo-Erba elemental analyzer interfaced with an Optima gas source mass spectrometer.

We corrected all tissues for the Suess effect, which is defined as the global decrease of ^13^C in Earth's atmospheric CO_2_, primarily due to fossil fuel burning over the past 150 years [42]–[44]. Based on ice core records [45], we applied a time-dependent correction of −0.022‰ per year [46] (to 2009) to all sample isotope values, except 2009 human hair.

### IsotopeR's model features

Unlike other SIMM models we incorporate all features currently used in SIMM analysis as well as other important features (Table 1). Appendix S1 describes IsotopeR features, illustrates how features interrelate, and defines prior distributions. For those interested, we also provide the model likelihood (Appendix S2). IsotopeR's structure is hierarchical (similar to the Semmens et al. model), such that an individual estimate is conditional on the group or population's distribution. The hierarchical structure of the model allows us to make statistical inference on each individual in the population, even though we only have one observation for each individual. Although we calculate individual estimates using only one observation, the structure of our model allows for repeated observations of the same individual. Including repeated measures for each individual consumer would result in less influence from the population-level and more accurate individual-level estimates.

**Figure 2:**
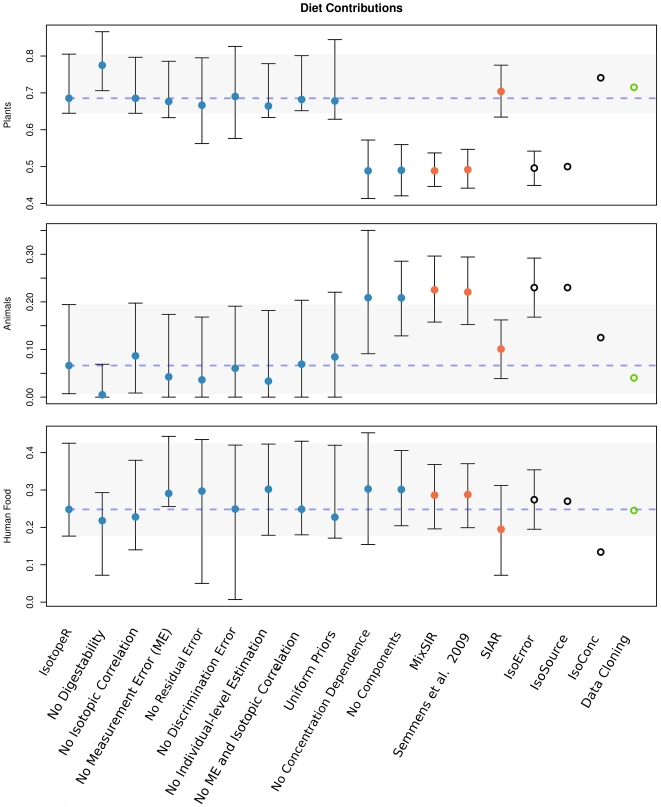
Model comparisons. Means and 95% credible intervals (denoted by error bars) calculated by IsotopeR (blue circles) and other Bayesian (orange circles) models. The blue dashed line and gray bar indicates the estimated mean and 95% credible interval for the full IsotopeR model, respectively. Frequentist (open black circles with confidence intervals) and data cloning estimates (open green circles) are also illustrated.

**Figure 3:**
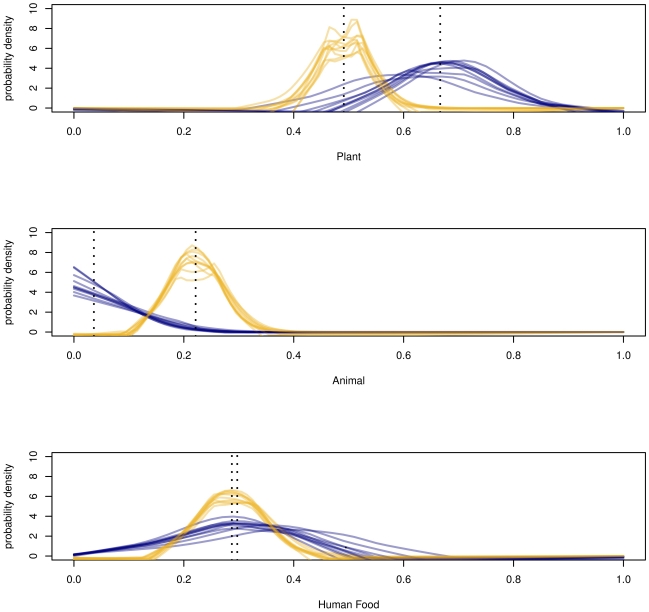
Dietary estimates generated by IsotopeR and the Semmens et al. model. Proportional dietary estimates (marginal posterior probability distributions) for individual bears (*n* = 11) estimated by IsotopeR (blue lines) and the Semmens et al. model (orange lines). Dotted lines denote population-level dietary estimates.

Whereas current SIMMs consider input parameters as known quantities, IsotopeR considers them random variables. Similar to Ward et al. (35), these variables are estimated using a fully Bayesian approach, which incorporates all the uncertainty associated with the joint estimation process. In our analysis, we jointly estimated 75 parameters using the full IsotopeR model. Incorporating the uncertainty associated with estimating multiple parameters leads to more accurate intervals [47] for sources and their concentrations. We reported 95% credible intervals, as well as means and standard deviations to illustrate (Fig. 1) and statistically summarize (Table 2B) our isotopic mixing space. In addition to defining our mixing space, we simultaneously estimated the joint posterior probability distribution of the sampled population's dietary source contributions. In the end, we reported marginal posterior distributions for each dietary source at the population- (Fig. 2, Table S5) and individual-level (Fig. 3, Table S6).

We follow the transformational procedure described by Semmens et al. [31] to estimate proportional diet contributions using Markov chain Monte Carlo (MCMC). This approach assumes that the observed isotopic distribution of an individual i and element e is a mixture distribution (M_i,e_) where the isotopic distribution of each source s (X_s,e_) is weighted by the individual's assimilated diet proportion(f_s,e,i_) of each element. For a study with n food sources, the individual's observed isotopic distribution is given by
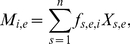
(2)where the vector of diet proportions for each element sums to 1, such that
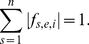
(3)Specifically, we assume that the vector of f_s_'s in equation 2 are random variables distributed using the centered log-ratio (CLR) transformation described by Semmens et al. [31]. This transformation allows us to use MCMC on the proportions in equation 3 on the continuous real line, and then transform results to the interval [0,1], resulting in estimators of proportions. Due to low acceptance rates, approaches such as importance sampling are difficult to apply when estimating numerous parameters. Therefore, we used a Gibbs sampler (a MCMC algorithm).

#### Isotopic correlation

Isotope ratios for different elements are often assumed to be independent because independent biochemical and ecological processes are ultimately responsible for their fractionation [24]. Although the processes explaining most of the variation in different elements may be different (e.g., photosynthetic pathway for carbon vs. trophic enrichment for nitrogen), secondary factors can lead to coupling between isotopic ratios of different elements [27], [40], [48], [49]. For example, several bear (Ursidae) studies that used SIA provided evidence that the nutritional pathways of carbon and nitrogen may be linked and the strength of correlation may increase with trophic level [13], [50], [51].

Ignoring correlations in a model's covariance structure can have effects on both point estimates [52] and their intervals [53]. Besides IsotopeR, IsoError is the only model that considers isotopic correlation in mixing model calculations [21]; however, we use a different approach to include this information in our estimation process. IsoError calculates the correlation coefficient (r) of the sources and the mixture and applies these values to correct the variance estimates. In contrast, we estimated r for all sources using Bayesian methods and included these estimates as terms in the covariance matrix (Appendix S1, #9).

#### Measurement Error

We estimated measurement error and applied it to each observation. Specifically, we measured this error from calibration runs used to ensure the mass spectrometer's accuracy. Because these calibrations are run on standards, we jointly estimated the measurement error (Appendix S1, #1, 2, 3) of the instrument along with the remaining model parameters.

#### Residual Error

We included a residual error term in our model to account for the error otherwise unaccounted for in the mixture. Our use of an error term (Appendix S1, #15,#25,#26) is consistent with standard linear regression models and is similar to other SIMMs (e.g., [30], [31]). This term takes into account unexplained variation, that is, variation not included in sources, discrimination processes, sub-sampling error, or measurement error.

#### Process and discrimination error

Differences between the isotope ratios in tissues of consumers and their dietary sources result from fractionation and stoichiometric effects (i.e., isotopic routing) [54]. In general, animal tissues are ^15^N- and ^13^C-enriched relative to their diets because lighter isotopes (^14^N, ^12^C) are preferentially eliminated via waste [6] and respiration [2], respectively, allowing heavier isotopes (^15^N, ^13^C) to be assimilated into animal tissues. These differences are commonly called “discrimination factors” and will vary depending on factors such as the taxon and tissue analyzed [55], a consumer's nutritional status (e.g., [56], [57]), sex [58], and the macromolecular composition of diet (e.g., [12], [23], [59]–[61]). Discrimination factors are often estimated (mean and SD) from results from controlled diet studies, and are used to shift food sources to consumers in an isotopic mixing space. These corrections are critical to accurately estimating proportional dietary contributions using SIMMs [23].

Discrimination factors extracted from the literature are assumed to be true and predicted correctly from regression models fitted to controlled diet data [55]. Using these fixed values can result in erroneous results when estimating mixed diets of free-ranging animals using SIMMs [62]. Recent research suggests that some controlled studies have used invalid procedures to predict discrimination factors [58], [61], [63]. For example, studies that fed captive bears controlled diets [50], [64] regressed tissue isotope values on food isotope values. The predicted discrimination factor for each natural food source was the difference between the isotope value of the food source and the predicted isotope value for the tissue; the latter calculated from entering the food isotope value into the regression model. Robbins et al. [61] note that regression coefficients calculated by such methods are biased at estimating discrimination factors because tissue isotope values (diet isotope value+discrimination factor) and diet isotope values (tissue isotope value – discrimination factor) are autocorrelated. Predicting discrimination factors using these covariates (in regression equations) yield spurious results; therefore, discrimination factors obtained by such methods should not be used to estimate the diets of animals using SIMM analysis. Furthermore, results from recent controlled diet studies using Sprague-Dawley rats suggest that correlations between discrimination factors and dietary isotope values are artifacts of the association between discrimination and biologically significant characteristics of diet (e.g., %N, % protein) that correlate with dietary isotope values. Therefore, if a regression approach is used, discrimination factors should be regressed on biologically significant characteristics of food, rather than food isotope values.

We used regression models developed by Kurle [58] to predict the tissue-diet discrimination factors of each sampled bear food. In this study, we defined discrimination factors as the differences between isotope values (δ^13^C and δ^15^N) for bear hair and sampled bear foods (expressed using Δ notation: Δ*X_tissue-diet_* = δ*X_tissue_*−δ*X_diet_*). Kurle [58] fitted regression models to data collected from a controlled diet study where omnivorous rats were fed various diets that equilibrated to their tissues. Because rats are often used as proxies for wild omnivores, we used the regression equations developed in Kurle [58] to predict discrimination factors for the hair of male bears on different % protein diets. Specifically, we entered the estimated % protein (x) of plant and animal foods—determined by multiplying %N of sampled foods by 6.25, or calculated from the NDB# (acorns only)—into the regression equations (Δ*^13^C* = *−0.14x+7.43*; Δ*^15^N* = *0.14x−2.10*) provided by Kurle [58] to predict Δ^13^C and Δ^15^N values for each sampled bear food. We then added each sample's Δ value to each sample's measured isotope value. Ultimately, the process error of the aggregate includes the inherent error associated with the isotopic variation of the samples in the aggregate and the variation of discrimination factors associated with each sample in the aggregate.

Current Bayesian models and some frequentist models allow users to apply fixed discrimination factors (predicted from regression equations, or extracted or inferred from the literature) and the associated uncertainty of each source to estimate dietary parameters. It is common for researchers to use discrimination factors from the literature instead of performing a complementary controlled experiment on their species of interest. Often researchers either use discrimination factors from a single controlled study that investigated discrimination in the same taxon or researchers use an average discrimination factor calculated from multiple studies (e.g., a waterfowl study calculated the mean discrimination factor from various controlled studies on birds). In addition to calculating the predicted discrimination factor for each plant and animal sample, we calculated the error (i.e., applied as a discrimination error term in the model; Appendix S1, #4) associated with the regression models used to predict these discrimination factors. Therefore, all known error associated with the discrimination process is accounted for in our model structure.

#### Concentration dependence

SIMMs that fail to account for stoichiometry in dietary sources may distort dietary estimates [26]. Including unequal elemental concentrations of sources when calculating dietary estimates using SIMMs will alter the polygonal isotopic mixing space, and in some cases, include mixtures that may have been previously outside the mixing space [24]. Similar to IsoConc [24] and SIAR [30], we strayed from the assumption that concentrations are equal among sources. Specifically, IsotopeR jointly estimated the concentrations (C and N) for each source (Table 2B) and incorporated the assimilation efficiency (i.e., digestibility) of different foods (Appendix S1, #10, 11, 12, 13, 14). We included the digestibility of each food source because previous studies [25], [26] suggest it is important to consider when incorporating concentration dependence in mixing model calculations. In particular, we estimated digestible [C] and [N] of human and bear foods by analyzing nutritional data from the NDB (Table S4) and sampled bear foods (Table S2), respectively. Calculations are described in Koch and Phillips [26] and in Table S2 and S4.

#### Aggregating plants and animals

We aggregated sampled bear foods into 3 sources: 100% plant diet, 100% animal diet, and 100% human food diet. We grouped acorns (n = 15), berries (n = 9), grass (n = 9), and forbs (n = 15) into a plant aggregate (n = 48), and deer (n = 5) and insects (n = 24) into an animal aggregate (n = 29) (Tables 2 & S3). We aggregated these natural food sources [38], [39] because they were biologically similar [65] and isotopically different (Table S2).

We used the three aggregated sources to estimate a joint probability distribution of proportional dietary source contributions for the sampled population and each individual bear. These distributions only provide inference to the foods we included in the model and will likely be biased, considering the omnivorous diets (i.e., they eat other plant and animal foods besides the species included in the analysis) of YNP black bears.

#### Prior distributions

The prior distribution can have an effect on inferences in Bayesian analysis. In particular, the prior can be especially influential when sample sizes are low; in such cases, using prior distributions derived from past results can improve inference [66]. Noninformative prior distributions (distributions that play a minimal role in the posterior distribution), also referred to as vague, flat, diffuse, or uninformative, are used in Bayesian analysis “to let the data speak for themselves, so that inferences are unaffected by information external to the current data” [66,61].

When conducting Bayesian analyses it is important to ascertain the influence of the prior on the posterior distribution; even when using noninformative priors. Likelihood methods such as data cloning may be used to examine such influence [67]. For each of the multivariate normal distributions in this study, we used a normal distribution prior to estimate mean parameters and gamma distributions for variance parameters. We assessed the effect priors had on inference by conducting a data cloning procedure described by Lele et al. [67]. For this procedure, we replicated the dataset (n = 10) and used these copies to swamp the posterior distribution, effectively minimizing the influence of the prior distribution [67]. Data cloning procedures yield estimator output that are asymptotically equivalent to maximum likelihood estimators. We evaluated the influence of prior distributions on our analysis by comparing the data cloning estimates to IsotopeR's estimates.

### Model comparisons

We calculated summary statistics for source aggregates and used them as input parameters in all models except IsotopeR (Tables 2A & 2C). We estimated the proportional source contributions (means and 95% credible intervals) for the sampled population using the full IsotopeR model and compared these estimates to those when each IsotopeR model feature was independently removed from the model (Fig. 2). In addition, we compared estimates by IsotopeR to estimates calculated by commonly used SIMMs (Fig. 2, Table S5). Lastly, we compared individual dietary estimates for bears calculated by IsotopeR to those calculated by the Semmens et al. model (Fig. 3).

Bayesian models have different convergence properties; therefore, we ran each model using a different number of iterations. We ran a burnin of 5×10^5^ draws for all IsotopeR models, followed by 15×10^5^ iterations of MCMC. We thinned our resulting chain by every 1,000 draws due to strong autocorrelation in some parameters. The Semmens et al. model used a burnin of 15×10^3^ draws, followed by 15×10^4^ iterations of MCMC that were thinned by every 100 draws, whereas SIAR was run at a burnin of 4×10^5^ draws, followed by 1×10^6^ iterations that were thinned by every 300 draws. MixSIR was run at a burnin of 5×10^3^ draws, followed by 3×10^4^ iterations.

## Results

### SIA and diet analysis

We analyzed the isotopic composition (δ^13^C, δ^15^N) of hair for 11 male FC black bears (Table S1) and estimated their diets using IsotopeR (Fig. 2, Table S5; Appendix S3). The protein content of sampled plants and animals were outside the bounds of the protein content in rat diets [58]; therefore, we extrapolated the discrimination factors used in this study. Specifically, the estimated protein content of sampled plants (range = 2.5–23.1%) was less than rat diets (range = 30–40%) and the estimated protein content of sampled animals (60.5–98.1%) was greater than rat diets (Table S2). Each predicted discrimination factor for each sample was added to the isotope value of each sample (Table S2). We used these adjusted values to estimate plant and animal source aggregates (Table S2). IsotopeR estimated all three sources (Table 2B) and the isotopic mixing space (Fig. 1). We note that source data (Tables 2A & 2C) and IsotopeR estimates for sources (Table 2B) were essentially equivalent.

**Figure 4:**
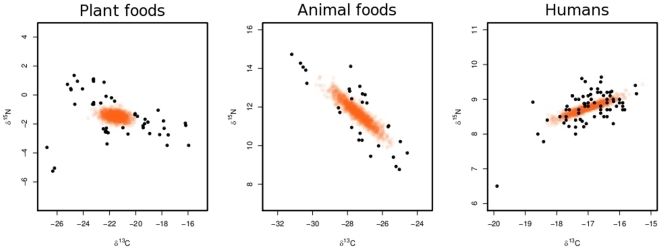
Isotopic correlation of δ^13^C and δ^15^N in each aggregated source. Orange circles indicate accepted draws from IsotopeR's MCMC chains; these values are used to estimate isotopic correlation and other source parameters. Black circles denote observed values.

IsotopeR estimated measurement error (δ^13^C: 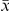 = 0.34; δ^15^N: 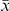 = 0.12) and applied this error to each observation. IsotopeR also included discrimination error (Δ^13^C = 1.96; Δ^15^N = 0.37) in its estimation process. We calculated isotopic correlation for use in IsoError (Table 2A) and IsotopeR estimated this relationship (Fig. 4, Table 2B). Animal and human δ^13^C and δ^15^N values were highly correlated (Figs. 4, Table 2) and all source correlations were similar to estimates calculated from the data (Tables 2A vs. 2B). We found that estimating correlation in the residual error term was unnecessary because the correlation in the bear population (r = 0.17) was accounted for by the correlation in the sources.

Estimated elemental concentrations among food sources were non-constant, causing the lines that connect the sources in the isotopic mixing space to be curvilinear (Fig. 1). Specifically, the isotopic data for animal matter had a higher [N] than sampled plants (t = 47.40, df = 47.12, P = <0.001; Table S2), regardless of whether digestibility corrections were included in the estimation (non-digest: t = 6.98, df = 47, P = <0.001; digest t = 9.96, df = 47.12, P = <0.001). As expected, ignoring the effect of concentration dependence among sources had a considerable effect on inference (Fig. 2, Table S5).

### IsotopeR features

We removed each feature from the model independently and compared inference to results from the full IsotopeR model (Fig. 2, Table S5). Removing correlation and measurement error independently had an effect on source estimates (especially for human food); although we note differences are similar to Monte Carlo error (∼3%). Removing the residual error term and discrimination error term (the latter independently having a larger effect) also had an effect on dietary estimates and increased the size of the credible intervals (Fig. 2, Table S5). Removing digestibility, concentration dependence, and all features separately from the full model had considerable influences on dietary estimates (Fig. 2, Table S5).

### Bayesian and frequentist SIMMs

Population estimates generated by IsotopeR, SIAR, and IsoConc were different than other estimates because these models included concentration dependence. In addition, the digestibility and non-digestibility population estimates for these models were different within and among models (Table S5). Results from the Semmens et al. model, MixSIR, IsoError, and IsotopeR without features (i.e., No components; Fig. 2) were all similar (Fig. 2, Table S5). Also, population estimates generated by the Semmens et al. model and MixSIR's were nearly identical (Fig. 2, Table S5); small differences in results were likely due to error associated with MCMC sampling and because the Semmens et al. model includes individual-level estimation.

Estimates by SIAR and IsotopeR were similar, yet slightly different. This difference was likely due to IsotopeR estimating dietary proportions at the individual-level; including isotopic correlation when estimating the mixing space; and accounting for the measurement error applied to each observation in the study and the error associated with a fully Bayesian approach. Including these important features will increase the accuracy of estimating dietary parameters.

IsotopeR's credible intervals for individuals were wider than estimates calculated by the Semmens et al. model (Fig. 3, Table S6). Mean estimates for human food were similar between models, but plant and animal proportions were different (Fig. 3, Table S6). This discrepancy was likely due to the fact that Semmens et. al. model did not include concentration dependence, measurement error, or a fully Bayesian approach. Furthermore, their model estimates were essentially the same for each individual (Fig. 3, Table S6), whereas IsotopeR provided a variety of dietary information for individuals (Table S6).

Point estimates by IsoError and IsoSource (tolerance of 0.05) were essentially identical; however, we note, IsoError provided confidence intervals and IsoSource did not. It is also important to note that mean estimates for these models were similar to all other models that did not include concentration dependence in their calculations.

### Influence of prior distributions

Data cloning and IsotopeR yielded similar dietary estimates (<3%) (Fig. 2, Table S5); therefore, we conclude that priors had little influence on the posterior distribution. We further tested the influence of the priors by changing all prior distributions to uniform distributions, which led to essentially no change (<3%) in our estimated population- or individual-level estimators (Fig. 2, Table S5). Given the Monte Carlo error present (∼3%) these results suggest that inferences are robust when using uninformative priors.

## Discussion

IsotopeR generated credible intervals that were generally wider than other models (Fig. 2, Tables S5 & S6); however, IsotopeR calculated more accurate parameter estimates because the model includes all recognized and quantifiable SIMM features, including measurement error, concentration dependence (with digestibility), isotopic correlation, individual-level estimation, and a fully Bayesian calculation. Collectively, these model features can have a considerable effect on dietary estimates when compared to commonly used models (Fig. 2, Tables S5 & S6).

Based on the analysis of our dataset, the Semmens et al. model, MixSIR, and IsoError, all generated very similar solutions (Fig. 2, Table S5). However, these models provide invalid estimates when elemental concentrations are nonconstant. Although IsoConc incorporates concentration dependence and had mean estimates similar to SIAR, like IsoSource, it does not calculate interval estimates. SIAR provides reasonable parameter estimates, but does not incorporate the sources of error and other important features IsotopeR includes in its model design.

### Measurement error, isotopic correlation, and residual error

We suggest SIMM users include measurement error in their estimation procedure because it exists, it can be estimated, and its absence in the estimation process can bias results (Fig. 2, Table S5). Previous studies have shown that not including measurement error may lead to biased parameter estimates and can also lead to a loss of statistical power [68]. We also found that accounting for measurement error increased the magnitude of correlation in sources. Not accounting for this error in measurements may effectively ‘wash out’ dependencies between variables and reduce estimates of isotopic correlation in sources.

It is important to account for isotopic correlation in sources because this relationship can affect the shape of the isotopic mixing space and the posterior probability distributions. Determining the proper shape of the mixing space is crucial when estimating the diets of animals using isotopic data. Although there may not always be enough measurements for source isotope values to accurately estimate correlation coefficients, our results suggest that including these estimates may be important when estimating the credible intervals of dietary proportions. In particular, evidence from our analysis suggests that the isotopic correlation of bears was explained by isotopic correlation in sources; however, future studies should determine if accounting for isotopic correlation in sources fully explains isotopic correlation in mixture data.

### Discrimination error

Isotopic discrimination is a complicated process and is difficult to accurately measure [23]. As a result, many researchers use discrimination factors from the published literature and assume they were estimated correctly. We corrected the isotope value for each food using a predicted discrimination factor and included the variability of these predictions in the estimation of source aggregates. In addition, we estimated sources using a discrimination error term, which represents the uncertainty associated with the regression models used to predict discrimination factors. Although our predicted discrimination factors are outside the regression range provided by Kurle [58], and are therefore unreliable, we assume interpolated predictions are valid and suggest researchers adjust each sample in their study in such a manner if feasible. We recommend sampling prey items to determine their nutrient compositions before deciding the range of biologically significant diets (e.g., ranging in protein quantity or quality [61]) to feed animals in a complementary controlled study. This will ensure regression models are useful in predicting discrimination factors for consumer's dietary sources.

We assumed rats, bears, and humans have similar discrimination factors since omnivorous species have similar digestive physiologies. Although this assumption is reasonable (i.e., rats are commonly used as a proxy for humans in controlled experiments), more controlled studies need to be conducted to determine if discrimination variation is negligible among omnivores on different protein quantity and quality diets.

### Concentration dependence

The assumption that elemental concentrations among sources are constant was violated and addressed in our analysis. Specifically, IsotopeR corrected the isotopic mixing space (Fig. 1) by accounting for digestible [C] and [N] values for each food source. When excluding this feature from the model, dietary estimates changed (Fig. 2, Table S5); a linear relationship between sources (inlay in Fig. 1) led to overestimated sources with greater N concentrations. Similar to other models that incorporate concentration dependence (i.e., SIAR and IsoConc), our full model estimates for plants increased considerably while animals and human food decreased. This occurred because estimated N concentrations were higher for animals and human food when compared to plants (Table 2). Correcting for differences in digestible C and N for source concentrations curved the lines that connected the isotopic endpoints and pinched the bottom of the mixing space. This decrease in area proximate to the plant aggregate increased the estimated proportion of plants to the diets of bears (Fig. 1). Although dietary estimates for omnivores are not reliable without taking concentration dependence (with digestibility corrections) into consideration, the effects of concentration dependence on SIMM inferences have not been evaluated using captive animals. Therefore, in addition to including concentration dependence in SIMM calculations it may also be important to exclude it from analysis and report all results.

### Greater than n+1 sources

Estimator coverage will decrease as the number of sources increase. This is due to the inability of the model to always estimate unique solutions when the number of sources is greater than the number of degrees of freedom (*n*+1). Therefore, we recommend reducing the amount of bias in SIMM analysis by having ≤n+1 sources. This can be accomplished by aggregating sources when they exceed n+1, adding dimensionality to the mixing space by including additional isotopes in the analysis, or eliminating sources that do not significantly contribute to the diets of animals as suggested by previous diet studies. Without taking one of these appropriate steps, a user will often calculate confounding results (i.e., inconsistent or bimodal posterior probability distributions). For example, a wolf population was partitioned into three groups and a Bayesian SIMM was used to make inferences about the diets of groups and individuals [31]. For the mainland group, the isotopic distribution of the sampled salmon population fell in the middle of the wolf distribution and directly between the deer and marine mammal distributions; this isotopic arrangement of sources confounded the estimation process. Adding another isotope (e.g., δ^34^S) or eliminating marine mammals from the analysis—only if they were shown in other studies to not contribute to the diets of wolves on the mainland—would have likely remedied this problem.

For omnivores, plant and animals may be aggregated into more groups (i.e., more dietary sources to estimate) if a user increases the number of isotopes used to make inference (e.g., including δ^34^S to estimate the contribution of salmon in diets of bears in Alaska). This would potentially increase the predictive power of the model [30], especially if sources were ≤n+1. It is important to put sufficient effort in using prior data (e.g., results from scat or gut content analysis) to determine the complete list of food sources and to aggregate them appropriately (e.g., [65]; as suggested in this study) to construct an isotopic mixing space that will produce unique and biologically significant solutions. In addition, such studies are also important when defining prior distributions in Bayesian SIMM analysis.

### Influence of prior distributions

Estimating all parameters simultaneously (i.e., fully Bayesian approach) is most useful when consumer sample size is low. When sample size increases, estimation error decreases, and parameter estimates will effectively become constants. Despite our small sample size (n = 11), data cloning point estimates were similar (<3%; Fig. 2, Table S5) to our model estimates; thus, suggesting the prior had little influenced on IsotopeR's parameter estimates.

### Conclusions

Here, we provide a review of commonly used SIMMs and offer a new comprehensive model. Our purpose was not to accurately estimate the real diets of YNP black bears. We used an incomplete collection of the plant foods and extrapolated discrimination factors; therefore, our dietary inferences are likely incorrect. However, we do believe our estimates are reasonable given what we know about the diets of FC bears in YNP and the nutrient requirements of bears. In particular, we believe it is reasonable for bears that regularly consume human food (18–43%), which is high in protein [41], to eat less animal matter (0–19%) than bears that do not consume human food. This is especially the case for YNP black bears since most of the animal matter in their diets is composed of insects [38], [39]. In addition, vegetation is clearly the largest contributor to the diets of bears (Fig. 2, Tables S5 & S6) as suggested by past diet studies conducted in YNP [38], [39].

SIMMs are evolving rapidly. We believe this expeditious process will result in the abandonment of many models currently used to estimate the diets of animals and the creation of many new models (e.g., time-series models). Because IsotopeR includes all features used in current models as well as other new features, we believe it will be the model of choice for many ecologists interested estimating the diets of animals using isotopic data. In addition, the model could be used as a foundation for future SIMM development because of its comprehensive structure; we note that IsotopeR, like other SIMMs, is also applicable for use in paleontology, archaeology, and forensic studies as well as estimating pollution inputs. The R package “IsotopeR” (with GUI) is available on CRAN (see R vignette and help files for directions on model use).

## Supporting Information

Table S1 **Suess-corrected isotope values for male food-conditioned black bears.** Year denotes the year the hair represents. Hair was Suess-corrected as described in Methods.
(DOC)

Table S2 **Adjusted isotopic data and digestibility calculations for sampled plants and animals.** Discrimination factors calculated for plant and animal aggregates derived from regression models in Kurle [58]. Digest [N] and [C] are calculated using the listed equations. Concentrations for *Quercus* spp. (acorns) were calculated using the USGS Nutrient Database (NDB) and other nutrition data [69]–[70].
(DOC)

Table S3 **Isotope values for human hair sampled from 2 salons and 1 barbershop in St. Louis, MO, 2009.**
(DOC)

Table S4 **Human food digestibility calculations for humans in the United States.** Three foods (minimum) were selected from each dietary source category provided by Nakamaru et al. [41]. Stoichiometric measurements were recorded for each food [located by entering each food's NDB# (Nutrient databank identifier) into the NDB search field (http://www.nal.usda.gov/fnic/foodcomp/search/)]. Digest [C] and Digest [N] are calculated using the listed formulas. Mean [C] and [N] are calculated for each source category and weighed according to the weighting factors (in parentheses next to each dietary source category; [41]). Weight [C] and [N] represent the weighted [C] and [N] for human food in the United States. These parameters are fixed and used to estimate proportional source contributions in all models that use such parameters.
(DOC)

Table S5 **Population-level dietary estimates generated by IsotopeR and commonly used SIMMs.**
(DOC)

Table S6 **Individual-level dietary estimates generated by IsotopeR and the Semmens et al. (2009) model.**
(DOC)

Appendix S1 **IsotopeR (full model) operational schematic.** Indented formulas on the left side denote terms and prior distributions associated with random variables. The right side provides a description of each formula. Subsection titles followed by the number of parameters estimated. Arrows denote hierarchical dependencies among random variables.
(DOC)

Appendix S2 **IsotopeR likelihood equation.**
(DOC)

Appendix S3 **Files and directions for running the full IsotopeR example used in this paper.**
(RAR)
