# Correction: Estimating the Diets of Animals Using Stable Isotopes and a Comprehensive Bayesian Mixing Model

**DOI:** 10.1371/annotation/d222580b-4f36-4403-bb1f-cfd449a5ed74

**Published:** 2012-01-12

**Authors:** John B. Hopkins, Jake M. Ferguson

There were errors in some formatting and symbols in two sentences of the Introduction text. The corrections are: "Isotope values (e.g., δX, δY) are expressed in delta (δ) notation as per mil (‰) units (or parts per thousand):" and "This system of three equations yields three unknown proportional source contributions (ƒ_1_, ƒ_2_, ƒ_3_) for a mixture (m) when δX and δY values are known for mixtures and sources (the latter adjusted to account for isotopic discrimination; described below)."

