# Supplementary material for: Estimating the Diets of Animals Using Stable Isotopes and a Comprehensive Bayesian Mixing Model
Source: PLoS One. 2012 Jan 3;7(1):e28478. doi: 10.1371/journal.pone.0028478 (PMC3250396; doi:10.1371/journal.pone.0028478)
Supplement: Table S1 — Suess-corrected isotope values for male food-conditioned black bears. Year denotes the year the hair represents. Hair was Suess-corrected as described in Methods. (DOC) [file pone.0028478.s001.doc]

| **ID** | **Year** | **δ^15^N** (‰) | **δ^13^C** (‰) | **Suess** (‰) | **Suess-corrected**  **δ^13^C** (‰) |
| --- | --- | --- | --- | --- | --- |
| 1 | 2005 | 4.57 | -20.54 | -0.088 | -20.63 |
| 2 | 2006 | 5.85 | -20.85 | -0.066 | -20.91 |
| 3 | 2005 | 3.90 | -22.30 | -0.088 | -22.38 |
| 4 | 2006 | 4.82 | -22.81 | -0.066 | -22.88 |
| 5 | 2006 | 3.45 | -22.00 | -0.066 | -22.06 |
| 6 | 2005 | 3.98 | -20.71 | -0.088 | -20.80 |
| 7 | 2007 | 3.74 | -20.36 | -0.044 | -20.41 |
| 8 | 2005 | 4.89 | -20.75 | -0.088 | -20.84 |
| 9 | 2007 | 4.00 | -22.12 | -0.044 | -22.17 |
| 10 | 2005 | 4.63 | -21.97 | -0.088 | -22.06 |
| 11 | 2006 | 4.21 | -22.38 | -0.066 | -22.44 |
| Mean |  | 4.37 |  |  | -21.60 |
| 1 SD |  | 0.68 |  |  | 0.88 |
