# Supplementary material for: Estimating the Diets of Animals Using Stable Isotopes and a Comprehensive Bayesian Mixing Model
Source: PLoS One. 2012 Jan 3;7(1):e28478. doi: 10.1371/journal.pone.0028478 (PMC3250396; doi:10.1371/journal.pone.0028478)
Supplement: Table S2 — Adjusted isotopic data and digestibility calculations for sampled plants and animals. Discrimination factors calculated for plant and animal aggregates derived from regression models in Kurle [58]. Digest [N] and [C] are calculated using the listed equations. Concentrations for Quercus spp. (acorns) were calculated using the USGS Nutrient Database (NDB) and other nutrition data [69]–[70]. (DOC) [file pone.0028478.s002.doc]

|  | **ID** | **δ^13^C (‰)** | **δ^13^C + Suess (‰)** | **δ^15^N**  **(‰)** | **% Protein** | **∆^15^N***  **(‰)** | **δ^15^N + ∆^15^N (‰)** | **∆^13^C***  **(‰)** | **δ^13^C + ∆^13^C (‰)** | **% C** | **% N** | **C:N** | **Digest**  **DM** | **Digest**  **N** | **Digest**  **C** | | **Digest**  **[N]** | **Digest**  **[C]** |
| --- | --- | --- | --- | --- | --- | --- | --- | --- | --- | --- | --- | --- | --- | --- | --- | --- | --- | --- |
|  | **Plant**  *Agrostis* sp. | -28.23 | -28.27 | -1.82 | 10.33 | -0.65 | -2.47 | 5.98 | -22.29 | 48.7 | 1.7 | 29.5 | 35 | 1.49 | 15.75 | | 4.25 | 45 |
|  | *Agrostis* sp. | -28.18 | -28.22 | -1.63 | 10.34 | -0.65 | -2.29 | 5.98 | -22.24 | 48.5 | 1.7 | 29.3 | 35 | 1.49 | 15.75 | | 4.25 | 45 |
|  | *Agrostis* sp. | -28.35 | -28.39 | -1.80 | 9.57 | -0.76 | -2.56 | 6.09 | -22.30 | 48.7 | 1.5 | 31.8 | 35 | 1.38 | 15.75 | | 3.94 | 45 |
|  | *Agrostis* sp. | -28.04 | -28.08 | 0.40 | 11.45 | -0.50 | -0.09 | 5.83 | -22.26 | 40.4 | 1.8 | 22.1 | 35 | 1.65 | 15.75 | | 4.71 | 45 |
|  | *Agrostis* sp. | -28.75 | -28.79 | 0.24 | 9.29 | -0.80 | -0.56 | 6.13 | -22.66 | 39.0 | 1.5 | 26.2 | 35 | 1.34 | 15.75 | | 3.82 | 45 |
|  | *Agrostis* sp. | -28.39 | -28.44 | 1.58 | 10.00 | -0.70 | 0.88 | 6.03 | -22.41 | 39.6 | 1.6 | 24.7 | 35 | 1.44 | 15.75 | | 4.12 | 45 |
|  | *Agrostis* sp. | -29.32 | -29.37 | 0.12 | 9.57 | -0.76 | -0.64 | 6.09 | -23.28 | 42.9 | 1.5 | 28.1 | 35 | 1.38 | 15.75 | | 3.94 | 45 |
|  | *Agrostis* sp. | -29.18 | -29.22 | 0.16 | 10.09 | -0.69 | -0.53 | 6.02 | -23.21 | 42.1 | 1.6 | 26.1 | 35 | 1.45 | 15.75 | | 4.15 | 45 |
|  | *Agrostis* sp. | -29.21 | -29.25 | 0.08 | 9.88 | -0.72 | -0.64 | 6.05 | -23.21 | 42.7 | 1.6 | 27.0 | 35 | 1.42 | 15.75 | | 4.07 | 45 |
|  | *Trifolium* spp. | -29.65 | -29.69 | -0.92 | 17.01 | 0.28 | -0.64 | 5.05 | -24.65 | 42.0 | 2.7 | 15.4 | 35 | 1.51 | 15.75 | | 4.30 | 45 |
|  | *Trifolium* spp. | -26.70 | -26.74 | -0.63 | 18.06 | 0.43 | -0.20 | 4.90 | -21.84 | 42.0 | 2.9 | 14.5 | 35 | 2.22 | 15.75 | | 6.35 | 45 |
|  | *Trifolium* spp. | -26.63 | -26.67 | -0.70 | 16.88 | 0.26 | -0.43 | 5.07 | -21.61 | 41.6 | 2.7 | 15.4 | 35 | 1.52 | 15.75 | | 4.34 | 45 |
|  | *Montia* spp. | -32.12 | -32.17 | -4.41 | 10.46 | -0.64 | -5.05 | 5.97 | -26.20 | 37.7 | 1.7 | 22.5 | 35 | 2.45 | 15.75 | | 7.00 | 45 |
|  | *Montia* spp. | -32.00 | -32.05 | -3.68 | 15.44 | 0.06 | -3.62 | 5.27 | -26.78 | 41.0 | 2.5 | 16.6 | 35 | 2.60 | 15.75 | | 7.43 | 45 |
|  | *Montia* spp. | -32.24 | -32.28 | -4.64 | 10.55 | -0.62 | -5.26 | 5.95 | -26.33 | 40.5 | 1.7 | 24.0 | 35 | 2.43 | 15.75 | | 6.95 | 45 |
|  | *Lupinus* spp. | -28.86 | -28.90 | 0.23 | 23.06 | 1.13 | 1.35 | 4.20 | -24.70 | 47.6 | 3.7 | 12.9 | 35 | 3.32 | 15.75 | | 9.49 | 45 |
|  | *Lupinus* spp. | -28.87 | -28.91 | -0.06 | 18.45 | 0.48 | 0.42 | 4.85 | -24.06 | 47.1 | 3.0 | 15.9 | 35 | 2.66 | 15.75 | | 7.59 | 45 |
|  | *Lupinus* spp. | -28.86 | -28.90 | 0.07 | 21.24 | 0.87 | 0.95 | 4.46 | -24.45 | 47.5 | 3.4 | 14.0 | 35 | 3.06 | 15.75 | | 8.74 | 45 |
|  | *Lupinus* spp. | -27.65 | -27.69 | 0.19 | 21.16 | 0.86 | 1.06 | 4.47 | -23.23 | 48.8 | 3.4 | 14.4 | 35 | 3.05 | 15.75 | | 8.71 | 45 |
|  | *Lupinus* spp. | -27.71 | -27.76 | 0.16 | 20.66 | 0.79 | 0.95 | 4.54 | -23.22 | 48.6 | 3.3 | 14.7 | 35 | 2.97 | 15.75 | | 8.50 | 45 |
|  | *Lupinus* spp. | -27.63 | -27.67 | 0.22 | 21.28 | 0.88 | 1.10 | 4.45 | -23.22 | 48.5 | 3.4 | 14.2 | 35 | 3.06 | 15.75 | | 8.76 | 45 |
|  | *Lupinus* spp. | -29.70 | -29.74 | -0.11 | 18.95 | 0.55 | 0.44 | 4.78 | -24.96 | 41.4 | 3.0 | 13.7 | 35 | 2.73 | 15.75 | | 7.80 | 45 |
|  | *Lupinus* spp. | -29.69 | -29.74 | -0.07 | 20.75 | 0.80 | 0.73 | 4.53 | -25.21 | 41.8 | 3.3 | 12.6 | 35 | 2.99 | 15.75 | | 8.54 | 45 |
|  | *Lupinus* spp. | -29.76 | -29.80 | -0.10 | 18.30 | 0.46 | 0.36 | 4.87 | -24.93 | 41.8 | 2.9 | 14.3 | 35 | 2.64 | 15.75 | | 7.53 | 45 |
|  | *Arctostaphylos* spp. | -25.58 | -25.62 | -1.31 | 5.25 | -1.37 | -2.67 | 6.70 | -18.93 | 52.9 | 0.8 | 63.0 | 63.4 | 0.76 | 28.53 | | 1.19 | 45 |
|  | *Arctostaphylos* spp. | -24.51 | -24.55 | -1.85 | 3.38 | -1.63 | -3.48 | 6.96 | -17.60 | 49.2 | 0.5 | 90.9 | 63.4 | 0.49 | 28.53 | | 0.77 | 45 |
|  | *Arctostaphylos* spp. | -25.21 | -25.25 | -0.99 | 2.50 | -1.75 | -2.74 | 7.08 | -18.17 | 51.2 | 0.4 | 127.8 | 63.4 | 0.36 | 28.53 | | 0.57 | 45 |
|  | *Arctostaphylos* spp. | -26.18 | -26.22 | -0.07 | 3.50 | -1.61 | -1.68 | 6.94 | -19.28 | 47.5 | 0.6 | 84.9 | 63.4 | 0.50 | 28.53 | | 0.79 | 45 |
|  | *Arctostaphylos* spp. | -26.20 | -26.24 | -0.67 | 4.54 | -1.46 | -2.13 | 6.79 | -19.44 | 46.8 | 0.7 | 64.4 | 63.4 | 0.65 | 28.53 | | 1.03 | 45 |
|  | *Arctostaphylos* spp. | -26.14 | -26.19 | -0.82 | 4.76 | -1.43 | -2.26 | 6.76 | -19.42 | 47.5 | 0.8 | 62.3 | 63.4 | 0.69 | 28.53 | | 1.08 | 45 |
|  | *Arctostaphylos* spp. | -27.49 | -27.53 | -1.03 | 6.17 | -1.24 | -2.26 | 6.57 | -20.97 | 44.0 | 1.0 | 44.6 | 63.4 | 0.89 | 28.53 | | 1.40 | 45 |
|  | *Arctostaphylos* spp. | -28.62 | -28.67 | -1.38 | 6.19 | -1.23 | -2.61 | 6.56 | -22.10 | 43.9 | 1.0 | 44.3 | 63.4 | 0.89 | 28.53 | | 1.41 | 45 |
|  | *Arctostaphylos* spp. | -28.73 | -28.77 | -1.33 | 6.14 | -1.24 | -2.57 | 6.57 | -22.20 | 43.6 | 1.0 | 44.4 | 63.4 | 0.88 | 28.53 | | 1.40 | 45 |
|  | *Quercus kelloggii* | -24.52 | -24.56 | -0.89 | 4.91 | -1.41 | -2.30 | 6.74 | -17.82 | 53.3 | 0.8 | 67.9 |  |  |  | | 0.64 | 52.31 |
|  | *Quercus kelloggii* | -24.70 | -24.75 | -0.25 | 3.98 | -1.54 | -1.79 | 6.87 | -17.87 | 48.1 | 0.6 | 75.5 |  |  |  | | 0.64 | 52.31 |
|  | *Quercus kelloggii* | -27.13 | -27.18 | -1.44 | 6.00 | -1.26 | -2.70 | 6.59 | -20.59 | 51.0 | 1.0 | 53.1 |  |  |  | | 0.64 | 52.31 |
|  | *Quercus kelloggii* | -26.85 | -26.89 | 0.13 | 4.34 | -1.49 | -1.36 | 6.82 | -20.07 | 51.2 | 0.7 | 73.7 |  |  |  | | 0.64 | 52.31 |
|  | *Quercus kelloggii* | -26.67 | -26.72 | 0.06 | 5.36 | -1.35 | -1.29 | 6.68 | -20.04 | 50.3 | 0.9 | 58.6 |  |  |  | | 0.64 | 52.31 |
|  | *Quercus kelloggii* | -24.97 | -25.01 | 0.35 | 5.00 | -1.40 | -1.05 | 6.73 | -18.28 | 45.9 | 0.8 | 57.3 |  |  |  | | 0.64 | 52.31 |
|  | *Quercus kelloggii* | -26.22 | -26.26 | -0.36 | 3.42 | -1.62 | -1.98 | 6.95 | -19.31 | 49.2 | 0.5 | 89.7 |  |  |  | | 0.64 | 52.31 |
|  | *Quercus kelloggii* | -25.78 | -25.83 | -0.45 | 4.75 | -1.44 | -1.88 | 6.77 | -19.06 | 46.6 | 0.8 | 61.4 |  |  |  | | 0.64 | 52.31 |
|  | *Quercus kelloggii* | -24.33 | -24.37 | -1.23 | 4.05 | -1.53 | -2.76 | 6.86 | -17.51 | 47.6 | 0.6 | 73.4 |  |  |  | | 0.64 | 52.31 |
|  | *Quercus wislizenii* | -22.81 | -22.85 | -1.92 | 3.83 | -1.56 | -3.48 | 6.89 | -15.96 | 45.1 | 0.6 | 73.7 |  |  |  | | 0.64 | 52.31 |
|  | *Quercus wislizenii* | -23.09 | -23.13 | -0.36 | 3.66 | -1.59 | -1.95 | 6.92 | -16.21 | 41.7 | 0.6 | 71.3 |  |  |  | | 0.64 | 52.31 |
|  | *Quercus wislizenii* | -22.91 | -22.95 | -0.65 | 4.63 | -1.45 | -2.10 | 6.78 | -16.17 | 41.9 | 0.7 | 56.7 |  |  |  | | 0.64 | 52.31 |
|  | *Quercus wislizenii* | -29.28 | -29.32 | -0.82 | 4.08 | -1.53 | -2.34 | 6.86 | -22.47 | 44.2 | 0.7 | 67.6 |  |  |  | | 0.64 | 52.31 |
|  | *Quercus wislizenii* | -26.62 | -26.67 | 0.00 | 4.50 | -1.47 | -1.47 | 6.80 | -19.87 | 42.8 | 0.7 | 59.5 |  |  |  | | 0.64 | 52.31 |
|  | *Quercus wislizenii* | -29.08 | -29.13 | -1.78 | 3.53 | -1.61 | -3.38 | 6.94 | -22.19 | 43.7 | 0.6 | 77.4 |  |  |  | | 0.64 | 52.31 |
|  | Mean | -27.49 | -27.53 | -0.75 | 9.82 | -0.73 | -1.48 | 6.06 | -21.47 | 45.4 | 1.57 | 44.24 |  |  |  | | 3.51 | 47.29 |
|  | 1 SD | 2.25 | 2.25 | 1.19 | 6.42 | 0.90 | 1.61 | 0.90 | 2.83 | 3.9 | 1.03 | 27.94 |  |  |  | | 3.09 | 3.43 |
|  | **Animal** |  |  |  |  |  |  |  |  |  |  |  |  |  |  | |  |  |
|  | *Odocoileus hemionus* | -24.49 | -24.53 | 2.72 | 94.90 | 11.19 | 13.91 | -5.86 | -30.39 | 45.2 | 15.2 | 2.98 | 100 | 15.18 | 51.50 | | 15.18 | 51.50 |
|  | *Odocoileus hemionus* | -23.99 | -24.03 | 1.59 | 98.09 | 11.63 | 13.23 | -6.30 | -30.33 | 48.4 | 15.7 | 3.08 | 100 | 15.69 | 51.50 | | 15.69 | 51.50 |
|  | *Odocoileus hemionus* | -25.26 | -25.30 | 3.56 | 91.51 | 10.71 | 14.27 | -5.38 | -30.69 | 43.0 | 14.6 | 2.93 | 100 | 14.64 | 51.50 | | 14.64 | 51.50 |
|  | *Odocoileus hemionus* | -25.43 | -25.47 | 3.67 | 93.95 | 11.05 | 14.73 | -5.72 | -31.19 | 43.4 | 15.0 | 2.89 | 100 | 15.03 | 51.50 | | 15.03 | 51.50 |
|  | *Odocoileus hemionus* | -25.41 | -25.45 | 3.65 | 89.42 | 10.42 | 14.07 | -5.09 | -30.54 | 41.6 | 14.3 | 2.91 | 100 | 14.31 | 51.50 | | 14.31 | 51.50 |
|  | Apidae | -24.73 | -24.77 | 4.11 | 74.41 | 8.32 | 12.43 | -2.99 | -27.76 | 43.3 | 11.9 | 3.64 | 100 | 11.91 | 51.50 | | 11.91 | 51.50 |
|  | Apidae | -24.72 | -24.77 | 4.47 | 75.44 | 8.46 | 12.93 | -3.13 | -27.90 | 43.3 | 12.1 | 3.59 | 100 | 12.07 | 51.50 | | 12.07 | 51.50 |
|  | Apidae | -24.72 | -24.76 | 4.39 | 75.27 | 8.44 | 12.82 | -3.11 | -27.87 | 43.4 | 12.0 | 3.60 | 100 | 12.04 | 51.50 | | 12.04 | 51.50 |
|  | *Camponotus* spp. | -23.47 | -23.52 | 1.12 | 80.46 | 9.16 | 10.29 | -3.83 | -27.35 | 48.9 | 12.9 | 3.80 | 100 | 12.87 | 51.50 | | 12.87 | 51.50 |
|  | *Camponotus* spp. | -23.50 | -23.55 | 1.32 | 78.99 | 8.96 | 10.28 | -3.63 | -27.17 | 49.4 | 12.6 | 3.91 | 100 | 12.64 | 51.50 | | 12.64 | 51.50 |
|  | *Camponotus* spp. | -23.49 | -23.53 | 1.57 | 79.79 | 9.07 | 10.64 | -3.74 | -27.28 | 49.7 | 12.8 | 3.89 | 100 | 12.77 | 51.50 | | 12.77 | 51.50 |
|  | *Camponotus* spp. | -23.89 | -23.94 | 1.39 | 72.55 | 8.06 | 9.45 | -2.73 | -26.67 | 50.2 | 11.6 | 4.32 | 100 | 11.61 | 51.50 | | 11.61 | 51.50 |
|  | *Camponotus* spp. | -23.87 | -23.92 | 2.62 | 63.40 | 6.78 | 9.40 | -1.45 | -25.37 | 51.7 | 10.1 | 5.10 | 100 | 10.14 | 51.50 | | 10.14 | 51.50 |
|  | *Camponotus* spp. | -23.43 | -23.48 | 3.18 | 60.94 | 6.43 | 9.62 | -1.10 | -24.58 | 43.7 | 9.75 | 4.49 | 100 | 9.75 | 51.50 | | 9.75 | 51.50 |
|  | *Camponotus* spp. | -23.36 | -23.40 | 3.31 | 64.25 | 6.89 | 10.21 | -1.56 | -24.97 | 43.6 | 10.3 | 4.24 | 100 | 10.28 | 51.50 | | 10.28 | 51.50 |
|  | *Lasius spp.* | -23.67 | -23.72 | 3.70 | 67.01 | 7.28 | 10.98 | -1.95 | -25.67 | 49.7 | 10.7 | 4.64 | 100 | 10.72 | 51.50 | | 10.72 | 51.50 |
|  | *Lasius spp.* | -23.72 | -23.76 | 3.83 | 66.46 | 7.20 | 11.03 | -1.87 | -25.64 | 49.7 | 10.6 | 4.67 | 100 | 10.63 | 51.50 | | 10.63 | 51.50 |
|  | Isoptera | -23.65 | -23.69 | 3.68 | 75.91 | 8.53 | 12.21 | -3.20 | -26.89 | 48.9 | 12.2 | 4.02 | 100 | 12.15 | 51.50 | | 12.15 | 51.50 |
|  | Isoptera | -25.26 | -25.31 | 3.26 | 75.46 | 8.46 | 11.72 | -3.13 | -28.44 | 48.6 | 12.1 | 4.02 | 100 | 12.07 | 51.50 | | 12.07 | 51.50 |
|  | Isoptera | -25.30 | -25.34 | 3.46 | 75.73 | 8.50 | 11.96 | -3.17 | -28.52 | 48.5 | 12.1 | 4.00 | 100 | 12.12 | 51.50 | | 12.12 | 51.50 |
|  | Isoptera | -25.14 | -25.18 | 3.62 | 60.50 | 6.37 | 9.99 | -1.04 | -26.22 | 56.1 | 9.7 | 5.80 | 100 | 9.68 | 51.50 | | 9.68 | 51.50 |
|  | Isoptera | -23.95 | -23.99 | 2.37 | 61.80 | 6.55 | 8.92 | -1.22 | -25.22 | 54.0 | 9.9 | 5.46 | 100 | 9.89 | 51.50 | | 9.89 | 51.50 |
|  | Isoptera | -23.88 | -23.93 | 2.33 | 60.94 | 6.43 | 8.77 | -1.10 | -25.03 | 53.7 | 9.8 | 5.51 | 100 | 9.75 | 51.50 | | 9.75 | 51.50 |
|  | Vespidae | -24.65 | -24.69 | 2.55 | 74.95 | 8.39 | 10.94 | -3.06 | -27.76 | 53.3 | 12.0 | 4.45 | 100 | 11.99 | 51.50 | | 11.99 | 51.50 |
|  | Vespidae | -23.71 | -23.75 | 4.20 | 78.33 | 8.87 | 13.07 | -3.54 | -27.29 | 49.8 | 12.5 | 3.98 | 100 | 12.53 | 51.50 | | 12.53 | 51.50 |
|  | Vespidae | -23.66 | -23.70 | 4.01 | 76.60 | 8.62 | 12.64 | -3.29 | -26.99 | 49.5 | 12.3 | 4.04 | 100 | 12.26 | 51.50 | | 12.26 | 51.50 |
|  | Vespidae | -23.83 | -23.88 | 3.65 | 76.47 | 8.61 | 12.25 | -3.28 | -27.15 | 49.9 | 12.2 | 4.08 | 100 | 12.23 | 51.50 | | 12.23 | 51.50 |
|  | Vespidae | -23.18 | -23.22 | 4.19 | 85.87 | 9.92 | 14.11 | -4.59 | -27.82 | 48.3 | 13.7 | 3.52 | 100 | 13.74 | 51.50 | | 13.74 | 51.50 |
|  | Vespidae | -23.89 | -23.94 | 4.14 | 75.96 | 8.53 | 12.68 | -3.20 | -27.14 | 50.8 | 12.2 | 4.18 | 100 | 12.15 | 51.50 | | 12.15 | 51.50 |
|  | Mean | -24.18 | -24.23 | 3.16 | 76.05 | 8.55 | 11.71 | -3.22 | -27.44 | 48.2 | 12.2 | 4.06 |  |  |  | | 12.17 | 51.50 |
|  | 1 SD | 0.71 | 0.71 | 1.00 | 10.55 | 1.48 | 1.74 | 1.48 | 1.82 | 3.8 | 1.7 | 0.77 |  |  |  | | 1.69 | 0.00 |
| Suess correction 2007-2009 = -0.022*2 = -0.044 | | | | | | | | | | | | | | | |  | | |
| % protein = % N x 6.25 | | | | | | | | | | | | | | | |  | | |
| Digest DM = compiled mean data [25] | | | | | | | | | | | | | | | |  | | |
| Digest N (plants) = % N x 0.9 (assuming 90% of % N is digestible)  Digest N (animals) = % N x 1 (assuming 100% of % N is digestible) | | | | | | | | | | | | | | | |  | | |
| Digest C (plants) = Digest DM x 0.45 (assuming Digest DM is 45% C for fruit and leafy plants; Koch and Phillips [26]) | | | | | | | | | | | | | | | |  | | |
| Digest C (animals) = 51.50 (determined by Phillips and Koch [24]) | | | | | | | | | | | | | | | |  | | |
| Digest [N] = (Digest N/Digest DM) x100 | | | | | | | | | | | | | | | |  | | |
| Digest [N] (acorns) = % protein dry weight (from NDB) x 0.16 N [70] x 0.466 (digestibility of pinenuts; [69])  = 8.53 x 0.16 x 0.466 = 0.64 | | | | | | | | | | | | | | | |  | | |
| Digest [C] = (Digest C/Digest DM) x100 | | | | | | | | | | | | | | | |  | | |
| Digest [C] (acorns) = (% protein dry weight (from NDB) x 0.52 C [70] x 0. 466 (digestibility of pinenuts [69]) + (% lipid dry weight x 0.75 C (100% digestible; [70]) + (% carbohydrate dry weight x 0.45 C (100% digestible; [70])  = (8.53 x 0.52 x 0.466) + (33.09 x 0.75) + (56.51 x 0.45) = 52.31 | | | | | | | | | | | | | | | |  | | |
| * discrimination factors derived from regression models [58] | | | | | | | | | | | | | | | |  | | |
