# Supplementary material for: Estimating the Diets of Animals Using Stable Isotopes and a Comprehensive Bayesian Mixing Model
Source: PLoS One. 2012 Jan 3;7(1):e28478. doi: 10.1371/journal.pone.0028478 (PMC3250396; doi:10.1371/journal.pone.0028478)
Supplement: Table S3 — Isotope values for human hair sampled from 2 salons and 1 barbershop in St. Louis, MO, 2009. (DOC) [file pone.0028478.s003.doc]

| **ID** | **δ^15^N** | **δ^13^C** |
| --- | --- | --- |
| 1 | 8.80 | -17.67 |
| 2 | 8.37 | -17.19 |
| 3 | 8.87 | -17.10 |
| 4 | 9.08 | -17.16 |
| 5 | 8.30 | -17.06 |
| 6 | 9.16 | -15.46 |
| 7 | 9.11 | -16.51 |
| 8 | 8.88 | -17.13 |
| 9 | 9.64 | -16.58 |
| 10 | 8.92 | -18.76 |
| 11 | 8.21 | -17.14 |
| 12 | 9.51 | -17.09 |
| 13 | 9.14 | -16.92 |
| 14 | 8.22 | -17.61 |
| 15 | 8.70 | -15.84 |
| 16 | 8.42 | -17.25 |
| 17 | 8.94 | -16.19 |
| 18 | 9.23 | -16.25 |
| 19 | 8.32 | -17.77 |
| 20 | 7.78 | -18.42 |
| Mean | 8.78 | -17.06 |
| 1 SD | 0.48 | 0.79 |
