# Supplementary material for: Estimating the Diets of Animals Using Stable Isotopes and a Comprehensive Bayesian Mixing Model
Source: PLoS One. 2012 Jan 3;7(1):e28478. doi: 10.1371/journal.pone.0028478 (PMC3250396; doi:10.1371/journal.pone.0028478)
Supplement: Table S4 — Human food digestibility calculations for humans in the United States. Three foods (minimum) were selected from each dietary source category provided by Nakamaru et al. [41]. Stoichiometric measurements were recorded for each food [located by entering each food's NDB# (Nutrient databank identifier) into the NDB search field (http://www.nal.usda.gov/fnic/foodcomp/search/)]. Digest [C] and Digest [N] are calculated using the listed formulas. Mean [C] and [N] are calculated for each source category and weighed according to the weighting factors (in parentheses next to each dietary source category; [41]). Weight [C] and [N] represent the weighted [C] and [N] for human food in the United States. These parameters are fixed and used to estimate proportional source contributions in all models that use such parameters. (DOC) [file pone.0028478.s004.doc]

| **Units = gm X/100 gm wet weight** | | | | | | | **Units = decimal % dry weight** | | | | **UNITS = gm X/100gm dry weight** | | | | | | | | | | | |
| --- | --- | --- | --- | --- | --- | --- | --- | --- | --- | --- | --- | --- | --- | --- | --- | --- | --- | --- | --- | --- | --- | --- |
| **Dietary source category/ NDB#** | **Water** | **Protein** | **Lipid** | **Carb.** | **Ash** | **Total** | **Protein** | **Lipid** | **Carb.** | **Ash** | **Protein**  **N** | **Digest**  **[N]** | **Protein**  **C** | | **Digest Protein C** | **Lipid**  **C** | **Carb.**  **C** | **Digest [C]** | **Weight [C]** | | **Weight [N]** | |
| Terrestrial meat (0.43) | | |  |  |  |  |  |  |  |  |  |  |  | |  |  |  |  |  | |  | |
| 13368 | 57.71 | 29.75 | 10.08 | 0.00 | 1.04 | 98.58 | 0.73 | 0.25 | 0.00 | 0.03 | 11.65 | 11.65 | 37.85 | | 37.85 | 18.50 | 0.00 | 56.35 |  | |  | |
| 13441 | 62.73 | 27.18 | 9.83 | 0.00 | 1.15 | 100.89 | 0.71 | 0.26 | 0.00 | 0.03 | 11.40 | 11.40 | 37.04 | | 37.04 | 19.32 | 0.00 | 56.36 |  | |  | |
| 13480 | 63.00 | 25.99 | 8.69 | 0.00 | 1.18 | 98.86 | 0.72 | 0.24 | 0.00 | 0.03 | 11.60 | 11.60 | 37.69 | | 37.69 | 18.17 | 0.00 | 55.86 |  | |  | |
| 10922 | 72.11 | 13.88 | 5.46 | 4.49 | 4.05 | 99.99 | 0.50 | 0.20 | 0.16 | 0.15 | 7.97 | 7.97 | 25.89 | | 25.89 | 14.69 | 7.25 | 47.82 |  | |  | |
| 10124 | 12.31 | 37.04 | 41.78 | 1.43 | 7.43 | 99.99 | 0.42 | 0.48 | 0.02 | 0.08 | 6.76 | 6.76 | 21.97 | | 21.97 | 35.74 | 0.73 | 58.44 |  | |  | |
| 10940 | 48.11 | 20.89 | 30.86 | 0.00 | 0.82 | 100.68 | 0.40 | 0.59 | 0.00 | 0.02 | 6.36 | 6.36 | 20.66 | | 20.66 | 44.03 | 0.00 | 64.69 |  | |  | |
| 05333 | 64.92 | 23.28 | 10.92 | 0.00 | 1.57 | 100.69 | 0.65 | 0.31 | 0.00 | 0.04 | 10.41 | 10.41 | 33.84 | | 33.84 | 22.90 | 0.00 | 56.74 |  | |  | |
| 05343 | 63.05 | 28.74 | 6.81 | 0.00 | 1.91 | 100.51 | 0.77 | 0.18 | 0.00 | 0.05 | 12.28 | 12.28 | 39.90 | | 39.90 | 13.63 | 0.00 | 53.53 |  | |  | |
| 05130 | 57.79 | 33.04 | 7.98 | 0.00 | 1.19 | 100.00 | 0.78 | 0.19 | 0.00 | 0.03 | 12.52 | 12.52 | 40.70 | | 40.70 | 14.18 | 0.00 | 54.88 |  | |  | |
| Mean |  |  |  |  |  |  |  |  |  |  |  | 10.10 |  | |  |  |  | 56.07 | 24.11 | | 4.34 | |
| 1 SD |  |  |  |  |  |  |  |  |  |  |  | 2.42 |  | |  |  |  | 4.42 |  | |  | |
| Fish/ Seafood (0.03) | | |  |  |  |  |  |  |  |  |  |  |  | |  |  |  |  |  | |  | |
| 15121 | 74.51 | 25.51 | 0.82 | 0.00 | 1.48 | 102.32 | 0.92 | 0.03 | 0.00 | 0.05 | 14.68 | 14.68 | 47.70 | | 47.70 | 2.21 | 0.00 | 49.91 |  | |  | |
| 15221 | 62.81 | 29.97 | 1.22 | 0.00 | 1.72 | 95.72 | 0.91 | 0.04 | 0.00 | 0.05 | 14.57 | 14.57 | 47.35 | | 47.35 | 2.78 | 0.00 | 50.13 |  | |  | |
| 15117 | 68.09 | 23.33 | 4.90 | 0.00 | 1.18 | 97.50 | 0.79 | 0.17 | 0.00 | 0.04 | 12.69 | 12.69 | 41.25 | | 41.25 | 12.50 | 0.00 | 53.75 |  | |  | |
| 15150 | 52.86 | 21.39 | 12.28 | 11.47 | 1.99 | 99.99 | 0.45 | 0.26 | 0.24 | 0.04 | 7.26 | 7.26 | 23.60 | | 23.60 | 19.54 | 10.95 | 54.09 |  | |  | |
| 15152 | 75.85 | 20.42 | 1.36 | 0.00 | 2.54 | 100.17 | 0.84 | 0.06 | 0.00 | 0.10 | 13.43 | 13.43 | 43.66 | | 43.66 | 4.19 | 0.00 | 47.86 |  | |  | |
| 15151 | 77.28 | 20.91 | 1.08 | 0.00 | 1.57 | 100.84 | 0.89 | 0.05 | 0.00 | 0.07 | 14.20 | 14.20 | 46.15 | | 46.15 | 3.44 | 0.00 | 49.59 |  | |  | |
| 15086 | 61.84 | 27.31 | 10.97 | 0.00 | 1.37 | 101.49 | 0.69 | 0.28 | 0.00 | 0.03 | 11.02 | 11.02 | 35.82 | | 35.82 | 20.75 | 0.00 | 56.57 |  | |  | |
| 35151 | 69.64 | 21.94 | 7.28 | 0.00 | 1.29 | 100.15 | 0.72 | 0.24 | 0.00 | 0.04 | 11.51 | 11.51 | 37.39 | | 37.39 | 17.90 | 0.00 | 55.29 |  | |  | |
| 15237 | 64.75 | 22.10 | 12.35 | 0.00 | 1.15 | 100.35 | 0.62 | 0.35 | 0.00 | 0.03 | 9.93 | 9.93 | 32.28 | | 32.28 | 26.02 | 0.00 | 58.30 |  | |  | |
| Mean |  |  |  |  |  |  |  |  |  |  |  | 12.14 |  | |  |  |  | 52.83 | 1.58 | | 0.36 | |
| 1 SD |  |  |  |  |  |  |  |  |  |  |  | 2.47 |  | |  |  |  | 3.60 |  | |  | |
| Milk/ Cheese (0.252) | |  |  |  |  |  |  |  |  |  |  |  |  | |  |  |  |  |  | |  | |
| 01211 | 88.13 | 3.15 | 3.27 | 4.78 | 0.67 | 100.00 | 0.27 | 0.28 | 0.40 | 0.06 | 4.25 | 4.25 | 13.80 | | 13.80 | 20.66 | 18.12 | 52.58 |  | |  | |
| 01084 | 88.74 | 3.93 | 1.17 | 5.52 | 0.86 | 100.22 | 0.34 | 0.10 | 0.48 | 0.07 | 5.48 | 5.48 | 17.80 | | 17.80 | 7.64 | 21.64 | 47.08 |  | |  | |
| 01085 | 90.84 | 3.37 | 0.08 | 4.96 | 0.75 | 100.00 | 0.37 | 0.01 | 0.54 | 0.08 | 5.89 | 5.89 | 19.13 | | 19.13 | 0.66 | 24.37 | 44.15 |  | |  | |
| 01009 | 36.75 | 24.90 | 33.14 | 1.28 | 3.93 | 100.00 | 0.39 | 0.52 | 0.02 | 0.06 | 6.30 | 6.30 | 20.47 | | 20.47 | 39.30 | 0.91 | 60.68 |  | |  | |
| 01040 | 37.12 | 26.93 | 27.80 | 5.38 | 2.77 | 100.00 | 0.43 | 0.44 | 0.09 | 0.04 | 6.85 | 6.85 | 22.27 | | 22.27 | 33.16 | 3.85 | 59.28 |  | |  | |
| 01146 | 25.00 | 37.86 | 27.34 | 3.41 | 6.39 | 100.00 | 0.50 | 0.36 | 0.05 | 0.09 | 8.08 | 8.08 | 26.25 | | 26.25 | 27.34 | 2.05 | 55.64 |  | |  | |
| Mean |  |  |  |  |  |  |  |  |  |  |  | 6.14 |  | |  |  |  | 53.24 | 13.42 | | 1.55 | |
| 1 SD |  |  |  |  |  |  |  |  |  |  |  | 1.29 |  | |  |  |  | 6.61 |  | |  | |
| Eggs (0.044) |  |  |  |  |  |  |  |  |  |  |  |  |  | |  |  |  |  |  | |  | |
| 01132 | 73.15 | 11.09 | 12.21 | 2.20 | 1.36 | 100.01 | 0.41 | 0.45 | 0.08 | 0.05 | 6.61 | 6.61 | 21.47 | | 21.47 | 34.09 | 3.69 | 59.25 |  | |  | |
| 01128 | 69.13 | 13.62 | 15.31 | 0.90 | 1.05 | 100.01 | 0.44 | 0.50 | 0.03 | 0.03 | 7.06 | 7.06 | 22.94 | | 22.94 | 37.18 | 1.31 | 61.43 |  | |  | |
| 01129 | 74.62 | 12.58 | 10.61 | 1.12 | 1.08 | 100.01 | 0.50 | 0.42 | 0.04 | 0.04 | 7.93 | 7.93 | 25.76 | | 25.76 | 31.34 | 1.99 | 59.09 |  | |  | |
| Mean |  |  |  |  |  |  |  |  |  |  |  | 7.20 |  | |  |  |  | 59.92 | 2.64 | | 0.32 | |
| 1 SD |  |  |  |  |  |  |  |  |  |  |  | 0.67 |  | |  |  |  | 1.31 |  | |  | |
| Legumes, nuts (0.03) | |  |  |  |  |  |  |  |  |  |  |  |  | |  |  |  |  |  | |  | |
| 11853 | 68.60 | 12.35 | 6.40 | 11.05 | 1.60 | 100.00 | 0.39 | 0.20 | 0.35 | 0.05 | 6.29 | 5.66 | 20.45 | | 18.41 | 15.29 | 15.84 | 49.53 |  | |  | |
| 11813 | 81.70 | 4.42 | 0.35 | 12.58 | 0.97 | 100.02 | 0.24 | 0.02 | 0.69 | 0.05 | 3.86 | 3.47 | 12.55 | | 11.29 | 1.43 | 30.90 | 43.62 |  | |  | |
| 16090 | 1.55 | 23.68 | 49.66 | 21.51 | 3.60 | 100.00 | 0.24 | 0.50 | 0.22 | 0.04 | 3.85 | 3.46 | 12.51 | | 11.26 | 37.83 | 9.83 | 58.92 |  | |  | |
| 12061 | 4.70 | 21.22 | 49.42 | 21.67 | 2.99 | 100.00 | 0.22 | 0.52 | 0.23 | 0.03 | 3.56 | 3.21 | 11.58 | | 10.42 | 38.89 | 10.23 | 59.55 |  | |  | |
| 12585 | 1.70 | 15.31 | 46.35 | 32.69 | 3.95 | 100.00 | 0.16 | 0.47 | 0.33 | 0.04 | 2.49 | 2.24 | 8.10 | | 7.29 | 35.36 | 14.96 | 57.62 |  | |  | |
| 12638 | 3.15 | 15.52 | 56.17 | 22.27 | 2.89 | 100.00 | 0.16 | 0.58 | 0.23 | 0.03 | 2.56 | 2.31 | 8.33 | | 7.50 | 43.50 | 10.35 | 61.34 |  | |  | |
| Mean |  |  |  |  |  |  |  |  |  |  |  | 3.39 |  | |  |  |  | 55.10 | 1.65 | | 0.10 | |
| 1 SD |  |  |  |  |  |  |  |  |  |  |  | 1.24 |  | |  |  |  | 6.96 |  | |  | |
| Cereals (0.135) | |  |  |  |  |  |  |  |  |  |  |  |  | |  |  |  |  |  | |  | |
| 11167 | 76.05 | 3.27 | 1.35 | 18.70 | 0.62 | 99.99 | 0.14 | 0.06 | 0.78 | 0.03 | 2.19 | 1.97 | 7.10 | | 6.39 | 4.23 | 35.15 | 45.77 |  | |  | |
| 20014 | 10.37 | 9.42 | 4.74 | 74.26 | 1.20 | 99.99 | 0.11 | 0.05 | 0.83 | 0.01 | 1.68 | 1.51 | 5.47 | | 4.92 | 3.97 | 37.29 | 46.17 |  | |  | |
| 19351 | 24.00 | 0.00 | 0.00 | 76.00 | 0.00 | 100.00 | 0.00 | 0.00 | 1.00 | 0.00 | 0.00 | 0.00 | 0.00 | | 0.00 | 0.00 | 45.00 | 45.00 |  | |  | |
| 18064 | 35.74 | 10.91 | 3.64 | 47.51 | 2.20 | 100.00 | 0.17 | 0.06 | 0.74 | 0.03 | 2.72 | 2.44 | 8.83 | | 7.95 | 4.25 | 33.27 | 45.46 |  | |  | |
| 20081 | 11.92 | 10.33 | 0.98 | 76.31 | 0.47 | 100.01 | 0.12 | 0.01 | 0.87 | 0.01 | 1.88 | 1.69 | 6.10 | | 5.49 | 0.83 | 38.98 | 45.30 |  | |  | |
| 08105 | 86.55 | 1.82 | 0.34 | 10.92 | 0.38 | 100.01 | 0.14 | 0.03 | 0.81 | 0.03 | 2.16 | 1.95 | 7.03 | | 6.33 | 1.89 | 36.51 | 44.73 |  | |  | |
| 20037 | 73.09 | 2.58 | 0.90 | 22.96 | 0.46 | 99.99 | 0.10 | 0.03 | 0.85 | 0.02 | 1.53 | 1.38 | 4.99 | | 4.49 | 2.51 | 38.41 | 45.41 |  | |  | |
| 20089 | 73.93 | 3.99 | 0.34 | 21.34 | 0.40 | 100.00 | 0.15 | 0.01 | 0.82 | 0.02 | 2.45 | 2.20 | 7.96 | | 7.16 | 0.98 | 36.84 | 44.98 |  | |  | |
| 20045 | 68.44 | 2.69 | 0.28 | 28.17 | 0.41 | 99.99 | 0.09 | 0.01 | 0.89 | 0.01 | 1.36 | 1.23 | 4.43 | | 3.99 | 0.67 | 40.18 | 44.83 |  | |  | |
| Mean |  |  |  |  |  |  |  |  |  |  |  | 1.60 |  | |  |  |  | 45.30 | 6.11 | | 0.10 | |
| 1 SD |  |  |  |  |  |  |  |  |  |  |  | 0.72 |  | |  |  |  | 0.47 |  | |  | |
| Potatoes (0.027) | |  |  |  |  |  |  |  |  |  |  |  |  | |  |  |  |  |  | |  | |
| 11403 | 62.48 | 2.66 | 5.22 | 27.74 | 1.90 | 100.00 | 0.07 | 0.14 | 0.74 | 0.05 | 1.13 | 1.02 | 3.69 | | 3.32 | 10.43 | 33.27 | 47.02 |  | |  | |
| 11829 | 75.42 | 1.96 | 0.10 | 21.55 | 0.97 | 100.00 | 0.08 | 0.00 | 0.88 | 0.04 | 1.28 | 1.15 | 4.15 | | 3.73 | 0.31 | 39.45 | 43.49 |  | |  | |
| 11875 | 75.78 | 2.01 | 0.15 | 20.71 | 1.35 | 100.00 | 0.08 | 0.01 | 0.86 | 0.06 | 1.33 | 1.20 | 4.32 | | 3.88 | 0.46 | 38.48 | 42.83 |  | |  | |
| Mean |  |  |  |  |  |  |  |  |  |  |  | 1.12 |  | |  |  |  | 44.45 | 1.20 | | 0.03 | |
| 1 SD |  |  |  |  |  |  |  |  |  |  |  | 0.09 |  | |  |  |  | 2.26 |  | |  | |
| Other Vegetables (0.054) | | |  |  |  |  |  |  |  |  |  |  |  | |  |  |  |  |  | |  | |
| 11109 | 92.18 | 1.28 | 0.10 | 5.80 | 0.64 | 100.00 | 0.16 | 0.01 | 0.74 | 0.08 | 2.62 | 2.36 | 8.51 | | 7.66 | 0.96 | 33.38 | 42.00 |  | |  | |
| 11124 | 88.29 | 0.93 | 0.24 | 9.58 | 0.97 | 100.01 | 0.08 | 0.02 | 0.82 | 0.08 | 1.27 | 1.14 | 4.13 | | 3.71 | 1.54 | 36.78 | 42.03 |  | |  | |
| 11090 | 89.30 | 2.82 | 0.37 | 6.64 | 0.87 | 100.00 | 0.26 | 0.03 | 0.62 | 0.08 | 4.22 | 3.80 | 13.70 | | 12.33 | 2.59 | 27.93 | 42.85 |  | |  | |
| Mean |  |  |  |  |  |  |  |  |  |  |  | 2.43 |  | |  |  |  | 42.29 | 2.28 | | 0.13 | |
| 1 SD |  |  |  |  |  |  |  |  |  |  |  | 1.33 |  | |  |  |  | 0.48 |  | |  | |
| Fruits (0.007) |  |  |  |  |  |  |  |  |  |  |  |  |  | |  |  |  |  |  | |  | |
| 09003 | 85.56 | 0.26 | 0.17 | 13.81 | 0.19 | 99.99 | 0.02 | 0.01 | 0.96 | 0.01 | 0.29 | 0.26 | 0.94 | | 0.84 | 0.88 | 43.07 | 44.79 |  | |  | |
| 09200 | 86.75 | 0.94 | 0.12 | 11.75 | 0.44 | 100.00 | 0.07 | 0.01 | 0.89 | 0.03 | 1.14 | 1.02 | 3.69 | | 3.32 | 0.68 | 39.91 | 43.91 |  | |  | |
| 09040 | 74.91 | 1.09 | 0.33 | 22.84 | 0.82 | 99.99 | 0.04 | 0.01 | 0.91 | 0.03 | 0.70 | 0.63 | 2.26 | | 2.03 | 0.99 | 40.98 | 44.00 |  | |  | |
| 09184 | 89.82 | 0.54 | 0.14 | 9.09 | 0.41 | 100.00 | 0.05 | 0.01 | 0.89 | 0.04 | 0.85 | 0.76 | 2.76 | | 2.48 | 1.03 | 40.18 | 43.70 |  | |  | |
| 09326 | 91.45 | 0.61 | 0.15 | 7.55 | 0.25 | 100.01 | 0.07 | 0.02 | 0.88 | 0.03 | 1.14 | 1.03 | 3.71 | | 3.34 | 1.31 | 39.69 | 44.34 |  | |  | |
| 09181 | 90.15 | 0.84 | 0.19 | 8.16 | 0.65 | 99.99 | 0.09 | 0.02 | 0.83 | 0.07 | 1.37 | 1.23 | 4.44 | | 4.00 | 1.45 | 37.32 | 42.76 |  | |  | |
| Mean |  |  |  |  |  |  |  |  |  |  |  | 0.82 |  | |  |  |  | 43.92 | 0.31 | | 0.01 | |
| 1 SD  Total  1 SD |  |  |  |  |  |  |  |  |  |  |  | 0.35 |  | |  |  |  | 0.68 | | 52.83  2.54 | | 6.88  1.10 |
| Factors for calculating macronutrient dry weight [70] | | | | | | | | | | | | | |  | | | | | | | | |
| Protein N = Protein (% dry weight) x 0.16 | | | | | | | | | | | | | |  | | | | | | | | |
| Protein C = Protein (% dry weight) x 0.52 | | | | | | | | | | | | | |  | | | | | | | | |
| Lipid C = Lipid (% dry weight) x 0.75 | | | | | | | | | | | | | |  | | | | | | | | |
| Carbohydrate C = Carbohydrate (% dry weight) x 0.45 | | | | | | | | | | | | | |  | | | | | | | | |
| ***Assume*** 100% digestibility for all sources for Lipid C and Carbohydrate C  ***Assume*** 100% protein digestibility for meat foods and 90% for plant foods [26] | | | | | | | | | | | | | |  | | | | | | | | |
| Digest Protein C (terrestrial meat, fish/seafood, milk/cheese, eggs) = Protein C x 1.0 | | | | | | | | | | | | | |  | | | | | | | | |
| Digest Protein C (legumes/nuts, cereals, potatoes, vegetables, and fruits) = Protein C x 9.0 | | | | | | | | | | | | | |  | | | | | | | | |
| Digest [C] = Digest Protein C + Lipid C + Carbo C | | | | | | | | | | | | | |  | | | | | | | | |
| Digest [N] (terrestrial meat, fish/seafood, milk/cheese, eggs) = Protein N x 1.0 | | | | | | | | | | | | | |  | | | | | | | | |
| Digest [N] (legumes/nuts, cereals, potatoes, vegetables, fruits) = Protein N x 9.0 | | | | | | | | | | | | | |  | | | | | | | | |
| Weight [C] & [N] = mean Total Digest [C] and Digest [N] for each group x group weight | | | | | | | | | | | | | |  | | | | | | | | |
