# Supplementary material for: Estimating the Diets of Animals Using Stable Isotopes and a Comprehensive Bayesian Mixing Model
Source: PLoS One. 2012 Jan 3;7(1):e28478. doi: 10.1371/journal.pone.0028478 (PMC3250396; doi:10.1371/journal.pone.0028478)
Supplement: Table S5 — Population-level dietary estimates generated by IsotopeR and commonly used SIMMs. (DOC) [file pone.0028478.s005.doc]

|  | Mean | | | Median | | | Credible Interval (0.025 – 0.0975) | | | | |
| --- | --- | --- | --- | --- | --- | --- | --- | --- | --- | --- | --- |
|  | Plant | Animal | Human | Plant | Animal | Human | Plant | Animal | | Human | |
| Full model:  IsotopeR  no digestibility  Data cloning | 0.685  0.797  0.715 | 0.066  0.014  0.040 | 0.248  0.189  0.245 | 0.725  0.775 | 0.106  0.005 | 0.328  0.218 | 0.645 – 0.805  0.706 – 0.866 | 0.007 – 0.194  0 – 0.069 | | 0.177 – 0.425  0.072 – 0.293 | |
| Full model without: |  |  |  |  |  |  |  |  | |  | |
| Isotopic correlation | 0.685 | 0.087 | 0.228 | 0.678 | 0.079 | 0.246 | 0.645 – 0.797 | 0.009 – 0.197 | | 0.140 – 0.380 | |
| Measurement error (ME) | 0.677 | 0.043 | 0.291 | 0.664 | 0.043 | 0.313 | 0.633 – 0.786 | 0 – 0.174 | | 0.256 – 0.444 | |
| ME & isotopic correlation | 0.682 | 0.069 | 0.249 | 0.674 | 0.062 | 0.264 | 0.562 – 0.831 | 0 – 0.203 | | 0 – 0.431 | |
| Residual error | 0.667 | 0.036 | 0.297 | 0.663 | 0.001 | 0.319 | 0.563 – 0.795 | 0 – 0.168 | | 0.050 – 0.435 | |
| Discrimination error | 0.690 | 0.061 | 0.249 | 0.685 | 0.052 | 0.265 | 0.576 – 0.826 | 0 – 0.191 | | 0.007 – 0.420 | |
| Concentration- dependence | 0.488 | 0.209 | 0.303 | 0.488 | 0.207 | 0.302 | 0.414 – 0.572 | 0.091 – 0.350 | | 0.154 – 0.453 | |
| No individual-level estimation | 0.664 | 0.031 | 0.302 | 0.656 | 0 | 0.328 | 0.633 – 0.779 | 0 – 0.182 | | 0.179 – 0.423 | |
| All features | 0.490 | 0.208 | 0.301 | 0.488 | 0.205 | 0.296 | 0.421 – 0.508 | 0.129 – 0.285 | | 0.204 – 0.406 | |
| *with* uniform priors | 0.708 | 0.095 | 0.197 | 0.697 | 0.089 | 0.216 | 0.579 – 0.844 | 0 – 0.233 | | 0 – 0.420 | |
| Bayesian models: |  |  |  |  |  |  |  |  | |  | |
| MixSIR | 0.489 | 0.225 | 0.286 | 0.488 | 0.226 | 0.287 | 0.446 – 0.537 | 0.158 – 0.296 | | 0.196 – 0.368 | |
| SIAR  no digestibility | 0.704  0.815 | 0.101  0.035 | 0.195  0.150 |  |  |  | 0.634 – 0.775  0.754 – 0.881 | 0.039 – 0.162  0 – 0.073 | | 0.072 – 0.312  0.048 – 0.242 | |
| Semmens et al. (2009) | 0.492 | 0.221 | 0.288 | 0.490 | 0.221 | 0.289 | 0.442 – 0.547 | 0.152 – 0.294 | | 0.199 – 0.370 | |
| Frequentist models: |  |  |  |  |  |  |  |  | |  | |
| IsoConc  no digestibility | 0.741  0.871 | 0.125  0.070 | 0.134  0.059 |  |  |  | Confidence Interval (0.025 – 0.0975) | | | | |
| IsoError | 0.496 | 0.230 | 0.274 |  |  |  | 0.449 – 0.542 | | 0.168 – 0.292 | | 0.195 – 0.354 |
| IsoSource | 0.50 | 0.23 | 0.27 |  |  |  |  | |  | |  |
