# Supplementary material for: Estimating the Diets of Animals Using Stable Isotopes and a Comprehensive Bayesian Mixing Model
Source: PLoS One. 2012 Jan 3;7(1):e28478. doi: 10.1371/journal.pone.0028478 (PMC3250396; doi:10.1371/journal.pone.0028478)
Supplement: Table S6 — Individual-level dietary estimates generated by IsotopeR and the Semmens et al. (2009) model. (DOC) [file pone.0028478.s006.doc]

| **Dietary Source** | **Bear #** | **1** | **2** | **3** | **4** | **5** | **6** | **7** | **8** | **9** | **10** | **11** |
| --- | --- | --- | --- | --- | --- | --- | --- | --- | --- | --- | --- | --- |
| **Plants** | **IsotopeR** | 0.668  0.721  0.613 – 0.814 | 0.618  0.680  0.556 – 0.788 | 0.705  0.750  0.659 – 0.844 | 0.675  0.726  0.623 – 0.821 | 0.716  0.761  0.672 – 0.845 | 0.693  0.741  0.644 – 0.831 | 0.698  0.744  0.649 – 0.837 | 0.657  0.710  0.604 – 0.802 | 0.700  0.743  0.654 – 0.837 | 0.677  0.732  0.625 – 0.818 | 0.691  0.745  0.645 – 0.839 |
|  | **Semmens model** | 0.484  0.481  0.405 – 0.562 | 0.469  0.473  0.358 – 0.553 | 0.494  0.492  0.422 – 0.576 | 0.482  0.482  0.402 – 0.559 | 0.500  0.495  0.433 – 0.596 | 0.491  0.488  0.422 – 0.579 | 0.495  0.492  0.426 – 0.578 | 0.480  0.480  0.394 – 0.561 | 0.491  0.488  0.426 – 0.575 | 0.485  0.483  0.412 – 0.563 | 0.490  0.487  0.420 – 0.569 |
| **Animals** | **IsotopeR** | 0.070  0.112  0.008 – 0.217 | 0.080  0.125  0.008 – 0.254 | 0.064  0.099  0.007 – 0.119 | 0.070  0.112  0.007 – 0.217 | 0.061  0.095  0.007 – 0.194 | 0.065  0.103  0.006 – 0.203 | 0.064  0.101  0.006 – 0.199 | 0.072  0.113  0.007 – 0.228 | 0.065  0.104  0.007 – 0.201 | 0.069  0.110  0.006 – 0.217 | 0.066  0.103  0.007 – 0.205 |
|  | **Semmens model** | 0.217  0.216  0.112 – 0.321 | 0.222  0.222  0.119 – 0.324 | 0.213  0.213  0.119 – 0.308 | 0.219  0.220  0.123 – 0.321 | 0.210  0.210  0.115 – 0.303 | 0.212  0.213  0.112 – 0.311 | 0.211  0.211  0.113 – 0.308 | 0.218  0.219  0.116 – 0.320 | 0.214  0.216  0.115 – 0.311 | 0.218  0.217  0.116 – 0.317 | 0.216  0.215  0.116 – 0.317 |
| **Human Food** | **IsotopeR** | 0.262  0.352  0.179 – 0.470 | 0.302  0.410  0.206 – 0.547 | 0.231  0.310  0.161 – 0.420 | 0.254  0.340  0.173 – 0.455 | 0.223  0.296  0.152 – 0.398 | 0.242  0.322  0.164 – 0.436 | 0.238  0.316  0.161 – 0.427 | 0.271  0.360  0.183 – 0.486 | 0.235  0.311  0.161 – 0.435 | 0.253  0.345  0.168 – 0.456 | 0.241  0.320  0.162 – 0.450 |
|  | **Semmens model** | 0.300  0.299  0.179 – 0.437 | 0.309  0.305  0.178 – 0.459 | 0.293  0.292  0.173 – 0.417 | 0.299  0.297  0.175 – 0.431 | 0.290  0.290  0.165 – 0.312 | 0.297  0.295  0.168 – 0.423 | 0.293  0.292  0.173 – 0.424 | 0.302  0.300  0.175 – 0.432 | 0.294  0.293  0.175 – 0.421 | 0.298  0.295  0.169 – 0.431 | 0.294  0.293  0.173 – 0.419 |
