# Supplementary material for: Estimating the Diets of Animals Using Stable Isotopes and a Comprehensive Bayesian Mixing Model
Source: PLoS One. 2012 Jan 3;7(1):e28478. doi: 10.1371/journal.pone.0028478 (PMC3250396; doi:10.1371/journal.pone.0028478)
Supplement: Appendix S1 — IsotopeR (full model) operational schematic. Indented formulas on the left side denote terms and prior distributions associated with random variables. The right side provides a description of each formula. Subsection titles followed by the number of parameters estimated. Arrows denote hierarchical dependencies among random variables. (DOC) [file pone.0028478.s007.doc]

**

**
