# Supplementary material for: Estimating the Diets of Animals Using Stable Isotopes and a Comprehensive Bayesian Mixing Model
Source: PLoS One. 2012 Jan 3;7(1):e28478. doi: 10.1371/journal.pone.0028478 (PMC3250396; doi:10.1371/journal.pone.0028478)
Supplement: Appendix S2 — IsotopeR likelihood equation. (DOC) [file pone.0028478.s008.doc]

(1)

(2)

(3)

(4)

(5)

(6)

Each line in the likelihood equation corresponds to a section in our model diagram (Supplemental Appendix I). Lines 1 – 3 correspond to the likelihood of the Mixtures section: line 1 gives the distribution of the observed mixtures; line 2 is the distribution of diet proportions for individuals; and line 3 is the distribution of the population diet proportions. The CLR function on lines 2 and 3 is the transformation of the random variable X and is given by . This expression is used to transform numbers on the continuous real line to the interval (0,1), constraining the sum of those variables to 1. Line 4, 5, and 6 correspond to the Source Concentrations section, Source Isotope Values section, and Measurement Error section, respectively.
